# Chromatin modifier HUSH co-operates with RNA decay factor NEXT to restrict transposable element expression

**DOI:** 10.1016/j.molcel.2022.03.004

**Published:** 2022-03-28

**Authors:** William Garland, Iris Müller, Mengjun Wu, Manfred Schmid, Katsutoshi Imamura, Leonor Rib, Albin Sandelin, Kristian Helin, Torben Heick Jensen

**Affiliations:** 1 Department of Molecular Biology and Genetics, Aarhus University, Aarhus, Denmark; 2 Biotech Research and Innovation Centre (BRIC), Faculty of Health and Medical Sciences, University of Copenhagen, Copenhagen, Denmark; 3 Novo Nordisk Foundation for Stem Cell Biology, Faculty of Health and Medical Science, University of Copenhagen, Copenhagen, Denmark; 4 The Bioinformatics Centre, Department of Biology, University of Copenhagen, Copenhagen, Denmark; 5 Cell Biology Program and Center for Epigenetics, Memorial Sloan Kettering Cancer Center, New York, NY, USA; 6 SciLifeLab, Department of Microbiology, Tumor and Cell Biology, Karolinska Institutet, Solna, Sweden; 7 Present address: The Institute of Cancer Research (ICR), London, UK; 8 Lead contact

## Abstract

Transposable elements (TEs) are widespread genetic parasites known to be kept under tight transcriptional control. Here, we describe a functional connection between the mouse-orthologous “nuclear exosome targeting” (NEXT) and “human silencing hub” (HUSH) complexes, involved in nuclear RNA decay and the epigenetic silencing of TEs, respectively. Knocking out the NEXT component ZCCHC8 in embryonic stem cells results in elevated TE RNA levels. We identify a physical interaction between ZCCHC8 and the MPP8 protein of HUSH and establish that HUSH recruits NEXT to chromatin at MPP8-bound TE loci. However, while NEXT and HUSH both dampen TE RNA expression, their activities predominantly affect shorter non-polyadenylated and full-length polyadenylated transcripts, respectively. Indeed, our data suggest that the repressive action of HUSH promotes a condition favoring NEXT RNA decay activity. In this way, transcriptional and post-transcriptional machineries synergize to suppress the genotoxic potential of TE RNAs.

## In brief

Garland et al. report a physical and functional connection between the NEXT complex, involved in RNA decay, and the HUSH complex, involved in chromatin regulation. Together, NEXT and HUSH cooperate to control transposable element (TE) RNA expression in embryonic stem cells, suppressing pA^−^ and pA^+^ transcripts, respectively.

## Graphical Abstract

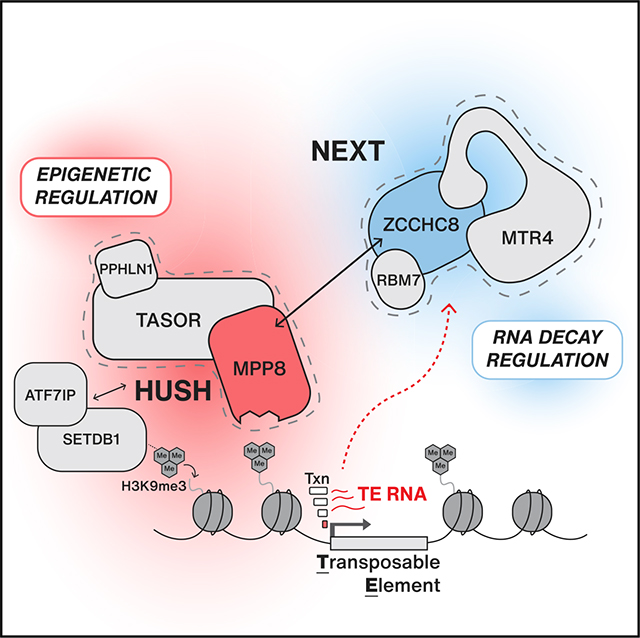

## INTRODUCTION

Mammalian genomes are colonized by transposable elements (TEs), which occupy as much as 50% of total genomic sequence and harbor the potential to propagate and reinsert themselves in a parasitic fashion (Cordaux and Batzer, 2009; Lander et al., 2001; Waterston et al., 2002). Such TE mobilization can lead to spontaneous mutation and therefore is subject to tight cellular control to maintain genome stability (Deniz et al., 2019; Goodier, 2016; Goodier and Kazazian, 2008). Although most described control mechanisms are epigenetically based, with DNA and histone modifications maintaining transcriptional repression through the formation of heterochromatin (Deniz et al., 2019; Slotkin and Martienssen, 2007), other exerting processes, both transcriptionally and post transcriptionally, have yet to be fully elucidated (Branco and Chuong, 2020).

The majority of mammalian TEs are classified as retrotransposons and replicate by transcribing an RNA intermediate that is reverse transcribed into cDNA before its genomic reintegration (Boeke et al., 1985). Retrotransposons can be divided into those harboring long terminal repeats (LTRs), such as endogenous retroviruses (ERVs), and non-LTR retrotransposons, including long- and short-interspersed nuclear elements (LINEs and SINEs, respectively). Although estimates suggest that most of these TEs are dormant because of recombination and/or mutation events gathered over time, active retrotransposons still occupy a considerable proportion of genomic sequence (Beck et al., 2010; Brouha et al., 2003; Khan et al., 2006; Mills et al., 2007). In somatic cells, retrotransposon expression is predominantly restricted by DNA methylation (Deniz et al., 2019; Molaro and Malik, 2016; Rollins et al., 2006; Smith et al., 2012). However, during early embryonic development, which can be modeled in embryonic stem cells (ESCs), DNA has significantly lower methylation levels compared to somatic cells, and, therefore, other mechanisms are employed to silence TE expression (Ghazimoradi and Farivar, 2020; He et al., 2019; Karimi et al., 2011; Matsui et al., 2010; Messerschmidt et al., 2014), including repressive histone modifications, such as H3K9me3. Here, a large family of Krüppel-associated box (KRAB) zinc finger proteins initially recognize TEs (Imbeault et al., 2017; Jacobs et al., 2014), which in turn attracts the adaptor protein TRIM28/KAP1 to act as a platform for the H3K9 methyltransferase SETDB1 and the H3K9me3-binding HP1 protein to build heterochromatin (He et al., 2019; Karimi et al., 2011; Matsui et al., 2010). This transcription-repressive environment is sustained around TE loci by other complexes, such as the human silencing hub (HUSH) complex, which is recruited by SETDB1 to aid in maintenance of the H3K9me3 modification. To exert this task, HUSH is composed of the chromodomain-containing protein MPP8 along with TASOR (FAM208A) and PPHLN1 (Periphilin) (Brummelkamp and van Steensel, 2015; Douse et al., 2020; Prigozhin et al., 2020; Tchasovnikarova et al., 2015; Timms et al., 2016), which together assist in silencing transcriptionally active TEs, including L1 LINEs and ERVs (Douse et al., 2020; Liu et al., 2018; Robbez-Masson et al., 2018; Seczynska et al., 2022).

Despite a strong focus on transcriptional regulators, specific examples from lower eukaryotes have demonstrated an important role for post-transcriptional mechanisms in TE suppression (Slotkin and Martienssen, 2007). Small RNA systems are major DNA silencers in flies, fission yeast, and nematodes and can act post transcriptionally, via RNA decay machineries, to facilitate the recruitment of heterochromatic factors to TEs (Andersen et al., 2017; Lee et al., 2013; Molaro and Malik, 2016). A prime example is the *S. pombe* Mtl1-Red1 core (MTREC) complex, which coordinates the decay of small RNAs produced from repetitive loci, such as centromeres, telomeres, and retrotransposons (Lee et al., 2013; Zofall et al., 2012). To do so, MTREC recruits the ribonucleolytic RNA exosome complex while, at the same time, promoting H3K9me3 deposition and heterochromatin assembly through its interaction with the RNA-induced transcriptional silencing (RITS) complex (Hiriart et al., 2012; Zofall et al., 2012). Therefore, MTREC illustrates a concerted action of transcriptional and post-transcriptional gene silencing that has yet to be revealed in higher eukaryotes despite the conservation of involved components (Meola et al., 2016; Silla et al., 2020). More recently, RNA modifications, such as *N*^6^-methyladenosine (m^6^A) and associated reader/writer proteins, have also been implicated in the regulation of TE expression in mammalian systems (Chelmicki et al., 2021; Li et al., 2020; Liu et al., 2020). However, the proposed findings are somewhat conflicting, and the underlying mechanisms require further investigation (He and Lan, 2021).

In mammalian nuclei, 3′−5′ exonucleolytic RNA decay is dominated by the exosome complex, which provides an essential layer of control to maintain stable transcriptomes (Mitchell et al., 1997; Schmid and Jensen, 2018). The exosome is involved in the management of the majority of nuclear RNAs and can function in productive processing events as well as in the complete removal of pervasively transcribed, aberrant, or otherwise nuclear retained transcripts (Garland and Jensen, 2020). To distinguish substrates, the exosome associates with so-called adaptors, providing target specificity. A fundamental component here is the RNA helicase MTR4 (SKIV2L2, MTREX), which is central to characterized nucleoplasmic adaptors; the “nuclear exosome targeting” (NEXT) complex and the “polyA tail exosome targeting” (PAXT) connection (Lubas et al., 2011; Meola et al., 2016; Silla et al., 2020). Both NEXT and PAXT promote exosomal decay of RNAPII transcripts aided by their connections to the cap-binding complex (Andersen et al., 2013; Giacometti et al., 2017; Winczura et al., 2018). NEXT is also composed of the zinc finger protein ZCCHC8 and the RNA-binding protein RBM7, which together drive decay of primarily short, unprocessed RNAs with non-adenylated 3′ ends (Gockert et al., 2022; Lubas et al., 2011, 2015; Wu et al., 2020a). PAXT, on the other hand, is comprised of a tight heterodimer between MTR4 and the zinc finger protein ZFC3H1, which, among other factors, associates with the nuclear poly(A)-binding protein PABPN1, suggested to target PAXT to primarily polyadenylated RNAs (Meola et al., 2016; Silla et al., 2020).

We have previously shown that both NEXT and PAXT activities are widespread, targeting RNAs produced from most, if not all, parts of actively transcribed chromatin (Lubas et al., 2015; Wu et al., 2020a, 2020b). However, these analyses only covered non-repetitive parts of the genome. Motivated by our earlier finding that RBM7 binds to TE transcripts in HeLa cells (Lubas et al., 2015) and that ZCCHC8 was recently proposed to target L1 LINE RNAs in early embryonic development (Wu et al., 2019), we set out to assess the contribution of NEXT and PAXT RNA decay pathways in the regulation of TE transcripts in mouse ESCs. Our results demonstrate that RNA decay plays a distinct role in restricting TE RNA expression and reveals a physical and functional connection between the NEXT and HUSH complexes. Although this connection provides the recruitment of NEXT to HUSH-bound TE loci, heterochromatic silencing by HUSH in parallel reduces transcription levels to favor conditions for NEXT-mediated decay. This reveals an unprecedented collaborative mechanism of transcriptional and post-transcriptional control to limit the genotoxic activity of TE RNAs in ESCs.

## RESULTS

### The NEXT complex affects TE RNA levels

**Figure 1.**
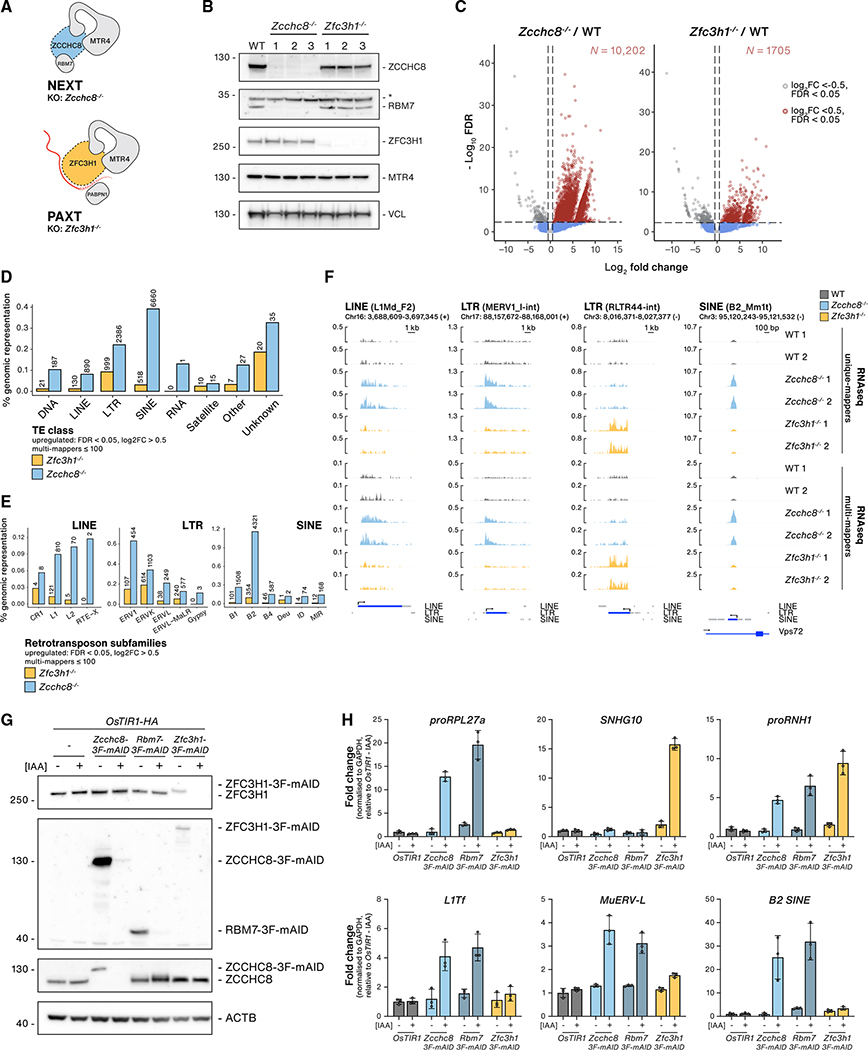
The NEXT complex impacts TE RNA levels (A) Schematic representations of the NEXT complex and the minimal PAXT connection. To inactivate these pathways, ZCCHC8 and ZFC3H1 loci were targeted using CRISPR-Cas9. (B) Western blotting (WB) analysis of wild-type (WT) and three independent *Zcchc8*^−/−^ and *Zfc3h1*^−/−^ clonal cell lines (1–3). Blots were probed with the indicated NEXT- and PAXT-related antibodies and Vinculin (VCL, loading control). Non-specific antibody signals are indicated with an asterisk (*). (C) DE analysis of TE RNAs from *Zcchc8*^−/−^ (left) or *Zfc3h1*^−/−^ (right) cells versus their WT control. The x axes show the average log_2_ FC of RNA-seq data, including multiple mappers ≤ 100, from three KO clones versus three WT samples, and y axes show the log_10_ false discovery rate (FDR) values. Vertical lines denote log_2_ FC = 0.5 or −0.5, and horizontal lines denote −log_10_ FDR = 1. Red dots denote significantly upregulated values (log_2_ FC > 0.5, FDR < 0.05), and gray dots denote significantly downregulated values (log_2_ FC < −0.5, FDR < 0.05). The number (N) of significantly upregulated TE RNAs are indicated for each KO condition in red font. (D) Bar plots of upregulated TE RNAs stratified by class (x axis). The y axis shows the percentage of significantly upregulated (log_2_ FC > 0.5, FDR < 0.05) RNAs relative to their genomic representation in *Zcchc8*^−/−^ and *Zfc3h1*^−/−^ versus control samples from multi-mapped (≤100) RNA-seq data. Absolute values of upregulated TE RNAs are indicated for each class. (E) As in (D) but stratified into retrotransposon subfamily classes. (F) Genome browser views of four upregulated TE RNA examples (LINE, LTR, SINE) from either unique or multi-mapped (≤100) RNA-seq data as indicated. RNAseq tracks from two replicates of stranded WT, *Zcchc8*^−/−^, and *Zfc3h1*^−/−^ samples are displayed with relevant strand direction (+/−) and genomic coordinates (mm10). TE annotations are extracted from the mouse Repeatmasker genomic dataset (mm10). Gene models are based on Gencode (M22). (G) WB analysis showing depletion of 3F-mAID-tagged proteins in OsTIR1-HA-expressing cells following −/+ treatment with IAA (12 h). Samples were derived from untagged, *Zcchc8–3F-mAID, Rbm7–3F-mAID*, and *Zfc3h1–3F-mAID* cells. Membranes were probed with antibodies against ZFC3H1, FLAG, ZCCHC8, and Actin (ACTB, loading control). (H) qRT-PCR analysis of indicated NEXT (*proRPL27a*), PAXT (*SNHG10*) or NEXT/PAXT (*proRNH1*) targets or TE RNAs (*L1Tf*, *MuERV-L*, *B2 SINE*) from total RNA isolated from cells described in (G). Results were normalized to *GAPDH* mRNA levels and plotted relative to OsTIR1-IAA control samples. Columns represent the average values of technical triplicates (individual data as points) with error bars denoting the SD.

To address a possible role of nuclear RNA decay pathways in the control of TE RNAs, we utilized mouse ESC lines disrupted for ZCCHC8 (NEXT) or ZFC3H1 (PAXT) expression through CRISPR-Cas9 engineering (Figure 1A). The generation of *Zfc3h1*^−/−^ cell lines was previously described (Garland et al., 2019), and equivalent *Zcchc8*^−/−^ cells were isolated from single-cell clones (Figure S1A), displaying strong co-depletion of the NEXT factor RBM7 (Hrossova et al., 2015; Mure et al., 2018; Tseng et al., 2015) (Figure 1B), while other associated factors remained unaffected (Figure S1B). Both *Zcchc8*^−/−^ and *Zfc3h1*^−/−^ cells were viable under 2i+LIF growth conditions, maintaining cultures at a level of so-called ground-state pluripotency (Ying et al., 2008) and showing normal mRNA expression of stem-cell-specific transcription factors *OCT4* and *SOX2* (Figure S1C). As a direct result of disrupting NEXT and PAXT pathways, *Zcchc8*^−/−^ and *Zfc3h1*^−/−^ cells displayed elevated levels of previously described nuclear exosome substrates, including PROMPTs, enhancer RNAs (eRNAs), and spliced snoRNA host gene (SNHG) long noncoding RNAs (Figure S1C). These transcripts demonstrated either NEXT (*proRPL27a*, *Nanog eRNA*, *proSNHG3*) or PAXT (*SNHG10*, *FAM120aos*, *proANKHD1*) specificity or sensitivity to both pathways (*proRNH1*) in line with observations of regional redundancy in human cells (Wu et al., 2020a).

For a global overview of changes in TE RNA expression, we interrogated sequencing data from rRNA-depleted total RNA samples from wild-type (WT) and *Zfc3h1*^−/−^ cells (Garland et al., 2019), which had been sequenced in parallel with *Zcchc8*^−/−^ samples. To focus on repetitive TEs, reads were re-mapped, allowing multiple alignments (≤100 multi-mappers), before quantification and differential expression (DE) analysis based on TElocal, a software package for analyzing TE expression that utilizes both unique and multi-mapped reads (Jin et al., 2015). In both *Zcchc8*^−/−^ and *Zfc3h1*^−/−^ cells, we observed significant (log_2_ fold change [FC] > 0.5, false discovery rate [FDR] < 0.05) upregulation of TE RNAs with a more pronounced phenotype in *Zcchc8*^−/−^ (N = 10,202) than in *Zfc3h1*^−/−^ samples (N = 1,705) (Figure 1C). Stratification of upregulated TE RNAs showed a strong effect on the three main classes of retrotransposons—LINEs, LTRs, and SINEs (Figure 1D)—using genomic representation to normalize for the relative abundance of each TE class. When examining absolute values and at a subfamily level, these mainly harbored L1 LINEs, murine endogenous retrovirus (MuERV) elements, and B2 SINEs (Figure 1E), which echoed similar observations in ESCs derived from *Zcchc8*^−/−^ blastocysts (Wu et al., 2019). Comparatively, *Zfc3h1*^−/ −^ cells exhibited an overall weaker effect, with LTR TE RNAs being the predominantly upregulated subtype (Figures 1D and 1E). These *Zcchc8*^−/−^ and *Zfc3h1*^−/−^ phenotypes were also evident when comparing normalized read counts aligning to retrotransposon classes in all samples (Figure S1D). The percentage of upregulated TEs in each class was <0.5% of genomic representation. This was highly significant considering the suggested fraction of active TEs (<1%) in mammalian genomes (Beck et al., 2010; Mills et al., 2007), which was also consistent with the percentage of TEs expressed above our cutoff filters (LINEs, 0.47%; LTRs, 0.75%; and SINEs, 1.2%). By scrutinizing genomic locations of upregulated elements, normalized to their genomic representation, we found that increased TE expression was widespread with a slight enrichment at coding sequence (CDS) features for both cell lines (Figure S1E). Although the use of multi-mappers is common practice in the analysis of repetitive elements, it also comes with caveats (see Limitations of the study). We therefore carried out the same analyses using only uniquely mapped reads and reassuringly obtained similar effects on the distribution of differentially expressed TE classes between cell lines (Figures S1F and 1D) and comparable phenotypes when scrutinizing individual loci (Figure 1F).

To validate our observations, we performed complementation experiments through exogenous expression of MYC-ZCCHC8 in *Zcchc8*^−/−^ cells, which emulated WT ZCCHC8 levels and rescued RBM7 levels (Figure S1G). Functional complementation was demonstrated by qRT-PCR analysis using primers against known NEXT-sensitive RNAs (Figure S1H, “PROMPTs”), and rescue was also recapitulated when interrogating TE RNAs using either generic primers against L1 or ERV-L family transcripts (*L1Tf*, *MuERV-L*) or locus-specific primers (*ABCB1a_L1*, *SORCS2_L1*) (Figure S1H, “TE RNAs”). Additionally, we generated conditional NEXT- and PAXT-depletion cell lines utilizing the auxin-inducible degron (AID) system to endogenously tag both alleles of RBM7, ZCCHC8 and ZFC3H1 loci (Natsume and Kanemaki, 2017). Tagged cell lines showed robust responses to auxin exposure using indole-3-acetic acid (IAA) and complete protein depletion was achieved in 1–2 h with an ensuing stabilization of known NEXT and PAXT targets (Figures S1I and S1J). After performing longer (12 h) depletions (Figure 1G), we analyzed RNA by qRT-PCR using primers for TE RNAs (Figure 1H). Consistent with the RNA sequencing (RNAseq) analysis of knockout (KO) cell lines, conditional depletion of NEXT components RBM7 and ZCCHC8 demonstrated upregulation of all tested TE RNAs in comparison to IAA or control cell lines, whereas depletion of ZFC3H1 showed a minor effect on *MuERV-L* (Figure 1H).

Taking all our analyses together, we conclude that NEXT and PAXT pathways are involved in the control of TE RNA expression in ESCs, with NEXT exhibiting the most prominent contribution.

### A physical and functional connection between the NEXT and HUSH complexes

**Figure 2.**
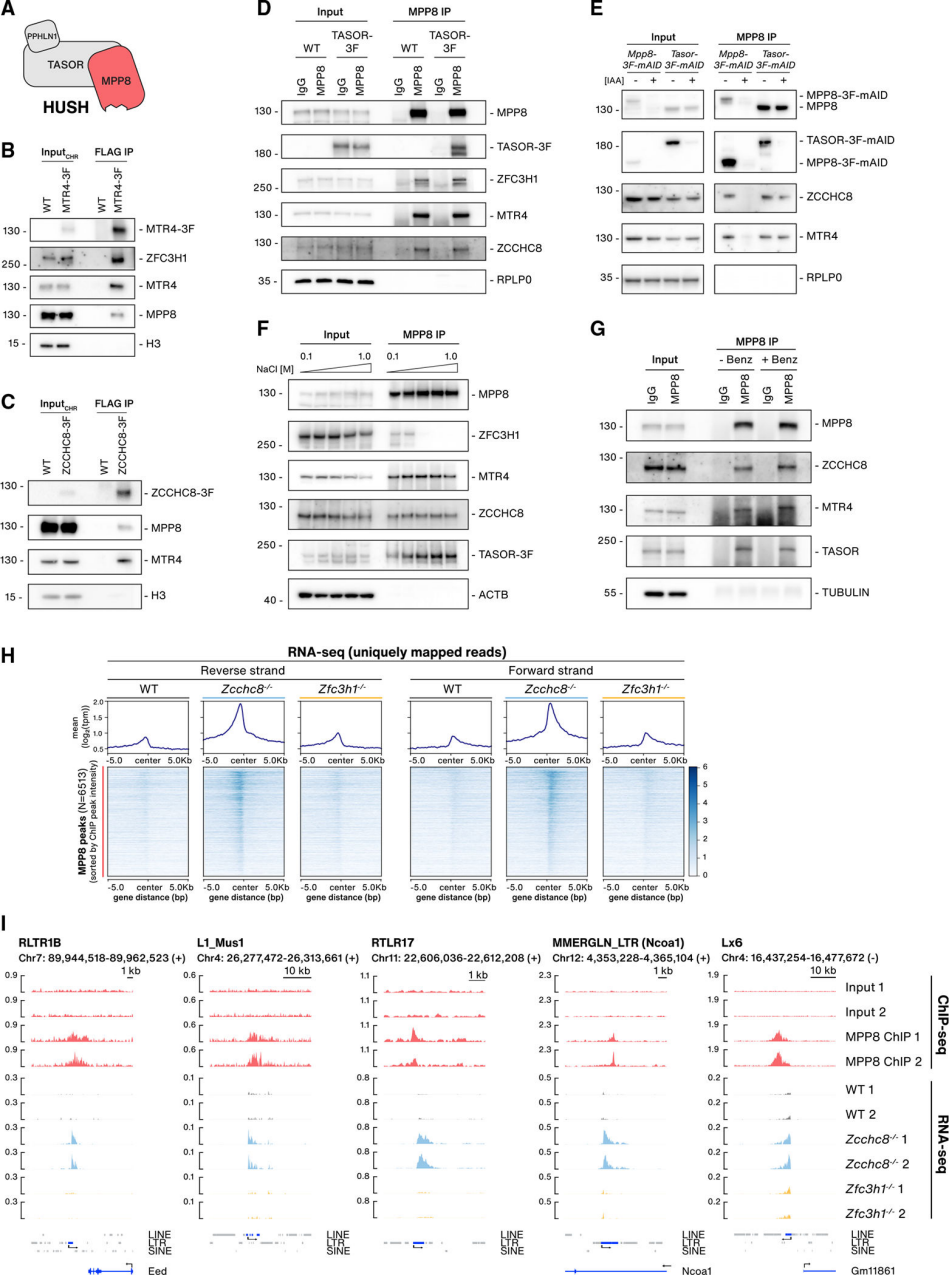
A physical and functional connection between the NEXT and HUSH complexes (A) Schematic representation of the HUSH complex. (B)WB analysis of FLAG IPs from chromatin lysates of WT and MTR4–3F cells. Chromatin input and IP samples were probed with antibodies against FLAG, ZFC3H1, MTR4, MPP8, and H3 (input loading control). (C) As in (B) but using *ZCCHC8–3F* cells. Membranes were probed with antibodies against FLAG, MPP8, MTR4, and H3 (input loading control). (D) WB analysis of MPP8 IPs from lysates of WT and TASOR-3F cells. IgG IPs were included as a negative control. Lysates from each cell line were split into two, with input samples loaded for each IP. Membranes were probed with antibodies against MPP8, FLAG, ZFC3H1, MTR4, ZCCHC8, and RPLP0 (input loading control). (E) WB analysis of MPP8 IPs from lysates of MPP8–3F-mAID or TASOR-3F-mAID cells either mock or IAA treated (8 h). Input and IP samples were probed with antibodies against MPP8, FLAG, ZCCHC8, MTR4, and RPLP0 (input loading control). (F) WB analysis of MPP8 IPs from lysates of TASOR-3F cells. Lysate extractions and IPs were carried out in increasing NaCl concentrations (0.1–1.0 M) as indicated. Membranes were probed with antibodies against MPP8, ZFC3H1, MTR4, ZCCHC8, FLAG, and Actin (ACTB, input loading control). (G) WB analysis of MPP8 IPs from WT lysates following mock or Benzonase treatment before final elution from beads. IgG IPs serve as a negative control. Lysates were split into two for either MPP8 or IgG IPs, with input samples loaded for each IP. Input and IP samples were probed with antibodies against MPP8, ZCCHC8, MTR4, TASOR, and TUBULIN (input loading control). (H) Metagene (upper) and heatmap (lower) profiles of unique mapped RNA-seq reads from WT, *Zcchc8*^−/−^, and *Zfc3h1*^−/−^ datasets within a 10 kb window centered on MPP8 ChIP peaks. Heatmap rows are sorted by MPP8 peak signal intensities. Coverage of uniquely mapped reads are displayed for + and − strands. (I) Genome browser views of five MPP8 target loci. Displayed tracks include input and MPP8 ChIP-seq data from two replicate experiments as well as RNA-seq data from two replicates of WT, *Zcchc8*^−/−^, and *Zfc3h1*^−/−^ samples. Strand directions (+/−) are noted along with genomic coordinates. TE hosting genes are indicated in parentheses.

We next speculated on possible recruitment mechanisms of NEXT/PAXT to TE loci and hypothesized that this might be mediated by chromatin-associated factors known to specifically associate with TEs. One such complex implicated in the regulation of LINEs and LTRs is the HUSH complex (Figure 2A), which is involved in SETDB1-dependent regulation of the H3K9me3 mark at LINE and LTR loci (Douse et al., 2020; Liu et al., 2018; Tchasovnikarova et al., 2015). More recently, HUSH was identified in a CRISPR screen for chromatin modifiers essential for maintaining ground-state pluripotency in ESCs, which suggested a vital role for this complex in suppression of hypomethylated TEs (Müller et al., 2021). With this in mind, we generated an endogenously tagged MTR4-3xFLAG (3F) line and carried out immunoprecipitation (IP) reactions from chromatin extracts. Along with known MTR4 interactors, we probed for the HUSH protein MPP8 and found that it co-precipitated with MTR4-3F (Figure 2B). In parallel experiments, employing analogously generated ZCCHC8-3F cells, MPP8 was also present in IP samples (Figure 2C). To support these observations, we performed reverse IPs using an MPP8 antibody and including TASOR-3F cells as an IP control. Reassuringly, the MPP8 antibody co-precipitated TASOR-3F as well as both MTR4 and ZCCHC8 (Figure 2D). We note that the PAXT component ZFC3H1 was also precipitated, suggesting that HUSH may interact with both NEXT and PAXT. Control IPs were carried out to ensure that the MPP8 antibody did not bind NEXT proteins non-specifically; MPP8 IPs were performed in conditional depletion conditions, utilizing generated *Mpp8-3F-mAID* and *Tasor-3F-mAID* cell lines, which demonstrated that ZCCHC8 and MTR4 were lost in MPP8 depletion samples but not in TASOR depletions (Figure 2E). These results also revealed insights into NEXT-HUSH contacts from the perspective of HUSH. As TASOR is reported to bridge the connection between MPP8 and PPHLN1 (Douse et al., 2020) (Figure 2A), our data implied that NEXT may interact directly with MPP8 or other MPP8-interacting proteins independent of the HUSH core.

To further characterize these interactions, MPP8 IPs were first carried out from TASOR-3F cell extracts in buffers with NaCl concentrations ranging from 100 mM to 1 M. Like TASOR-3F, both MTR4 and ZCCHC8 were retained in all IP conditions, demonstrating a high-affinity salt-resistant physical interaction (Figure 2F). In contrast, the ZFC3H1 interaction was lost in buffers containing >300 mM NaCl. The MPP8-ZCCHC8/MTR4 interaction was also resistant to Benzonase treatment (Figure 2G), which efficiently eluted the RNA-dependent MTR4 interactor PABPN1 in a control experiment (Figure S2A). Hence, the HUSH-NEXT interaction does not require an RNA or DNA intermediate. Finally, we fractionated cell lysates by ultracentrifugation through 10%–50% glycerol gradients, revealing co-sedimentation of NEXT components (MTR4, ZCCHC8, and RBM7) in high-molecular-weight fractions (Figure S2B, fractions 9–18). Blotting for HUSH components (MPP8, TASOR, PPHLN1) showed their co-fractionation with NEXT proteins (Figure S2B, fractions 5–18), although MPP8 was also found in lighter-molecular-weight fractions (Figure S2B, fractions 4–8), presumably owing to the presence of MPP8 in other protein complexes (Chang et al., 2011; Kokura et al., 2010; Sun et al., 2015).

Having established a tight and RNA-independent physical interaction between HUSH and NEXT, we pursued further relationships between the two complexes by first conducting chromatin IP sequencing (ChIP-seq) of MPP8 to determine HUSH locations genome-wide and with the aim to address any overlap with TE loci exhibiting ZCCHC8-sensitive RNA expression. Using MPP8 ChIP peaks, we displayed uniquely mapped RNA-seq read intensities from WT, *Zcchc8*^−/−^, and *Zfc3h1*^−/−^ samples at these regions (Figure 2H). Centered on such MPP8 peaks, a pronounced increase in RNA expression (log2 FC > 0.5, FDR < 0.05) was observed at 53% of these regions in *Zcchc8*^−/−^ compared with WT samples (Figure 2H), which is also illustrated by individual examples (Figure 2I). Incomparison, 4.6% of MPP8 peaks overlapped with increased RNA-seq signals in *Zfc3h1*^−/−^ samples (Figure 2H). To further dissect MPP8-bound regions, we stratified MPP8 ChIP peaks by their overlap with TE or non-TEloci and found that the majority overlapped with TEs (Figure S2C), with a particular enrichment at LTRs. Plotting RNA-seq signals at MPP8-bound TE loci further demonstrated a marked increase in *Zcchc8*^−/−^ compared with WT or *Zfc3h1*^−/−^ conditions (Figure S2D). To address the percentage of NEXT/PAXT-sensitive TEs that are also MPP8 bound, we intersected our RNA-seq data with MPP8-bound TEs (Figure S2E). With the applied cutoffs, we found that MPP8-bound TEs overlapped with ~25% of upregulated LTRs and ~10% of upregulated LINEs in *Zcchc8*^−/−^ samples with much lower numbers in *Zfc3h1*^−/−^ samples (Figures S2E and 1D). The overlap of MPP8 peaks with upregulated SINEs was particularly low (~3% in *Zcchc8*^−/−^ cells), echoing that HUSH is not involved in SINE regulation (Müller et al., 2021). We conclude that RNAs from HUSH-bound TEs show a high degree of sensitivity to NEXT depletion. Because HUSH-bound areas only cover a proportion of TEs upregulated in *Zcchc8*^−/−^ cells, other recruitment mechanisms of NEXT to TEs, such as SINEs, may exist (see Discussion).

We have previously reported a pervasive role for NEXT and PAXT in exosome-mediated decay of nuclear RNAs (Lubas et al., 2015; Wu et al., 2020a, 2020b). To address whether HUSH would be involved in NEXT or PAXT function outside of TE regulation, we also interrogated non-TE regions with RNAs upregulated (log2 FC > 0.5, FDR < 0.05) in *Zcchc8*^−/−^ (N = 1,567) or *Zfc3h1*^−/−^ (N = 1,540) samples. In both cases, the majority of regions (~85%) did not overlap with MPP8 ChIP peaks (Figure S2F; data not shown). We therefore conclude that HUSH is not required for NEXT and PAXT to function in the decay of RNAs from non-TE loci. That said, the widespread presence of NEXT-sensitive TE RNAs expressed from MPP8-bound loci, along with the physical HUSH-NEXT interaction, strongly suggested a functional connection between these complexes in repressing a subset of TEs. As the relation between HUSH and PAXT appeared less robust, we focused subsequent efforts on the HUSH-NEXT connection.

### ZCCHC8 bridges the interaction between NEXT and HUSH

**Figure 3.**
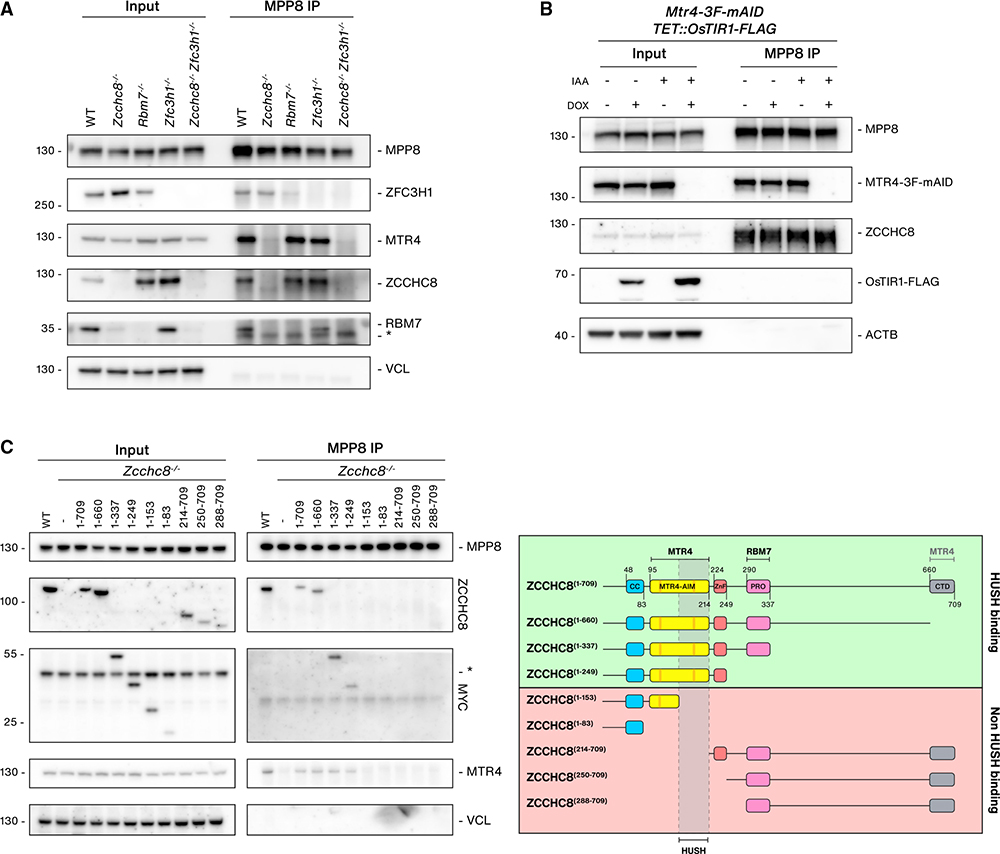
ZCCHC8 bridges the interaction between NEXT and HUSH (A) WB analysis of MPP8 IPs from lysates of WT, Z*cchc8*^−/−^, *Rbm7*^−/−^, *Zfc3h1*^−/−^, and *Zcchc8*^−/−^*Zfc3h1*^−/−^ cells. Input and IP samples were probed with antibodies against HUSH-, NEXT-, and PAXT-related proteins as indicated and Vinculin (VCL, input loading control). Non-specific bands are indicated with an asterisk (*). (B) WB analysis of MPP8 IPs from *TET*::*OsTIR1*-*FLAG*, *MTR4*-*3F*-*mAID* cells following doxycycline (DOX) and/or IAA treatment (4 h) as indicated. Input and IP samples were probed with antibodies against MPP8, MTR4, ZCCHC8 FLAG, and Actin (ACTB, input loading control). (C) Left: WB analysis of MPP8 IPs from WT or *Zcchc8*^−/−^ cells stably expressing MYC-tagged ZCCHC8 fragments labeled with amino acid numbers as in the right panel. Input and IP samples were probed with antibodies against MPP8, ZCCHC8, MYC, MTR4, and Vinculin (VCL, input loading control). Right: schematic representation of ZCCHC8 domains, generated fragments, and MPP8 IP data summary. Known protein binding regions are indicated on the top. Fragments shown to be HUSH binding (green) or not (red) are indicated, and a putative binding region is shown.

To explore the proteins responsible for connecting NEXT to HUSH, we generated additional KO cell lines. As RBM7 stability is compromised in *Zcchc8*^−/−^ cells, (Figure 1B), we generated *Rbm7*^−/−^ cell lines to distinguish the contribution of either NEXT component to the HUSH interaction. Contrary to ZCCHC8 depletion, RBM7 depletion does not affect ZCCHC8 stability (Hrossova et al., 2015; Lubas et al., 2011), which was recapitulated in the *Rbm7*^−/−^ cell line (Figure 3A). We also generated double *Zcchc8*^−/−^; *Zfc3h1*^−/−^ cell lines to determine whether the HUSH interaction is solely mediated by MTR4, which is central for both NEXT and PAXT. Conducting MPP8 IPs in these KO cell lines, we could demonstrate an MPP8 interaction with all three NEXT components, but it was subsequently lost in *Zcchc8*^*−*/*−*^ conditions (Figure 3A). Because both ZCCHC8 and MTR4 interactions with MPP8 remained in *Rbm7*^*−*/*−*^ cells, these analyses suggested that the HUSH-NEXT connection is mediated by ZCCHC8. We also noted that ZFC3H1 depletion had little effect on the interaction between MPP8 and MTR4, supporting the notion that the major connection between HUSH and MTR4 is via NEXT rather than PAXT (Figure 3A). Finally, to unequivocally address whether the HUSH-NEXT connection was solely mediated by ZCCHC8, we generated a conditional *MTR4*-*3F*-*mAID* cell line, which negates the previously observed long-term co-depletion effects of ZCCHC8 (Lubas et al., 2011) (Figure 3B). In the absence of MTR4, ZCCHC8 was still present in MPP8 IPs, and we therefore conclude that ZCCHC8 mediates the interaction between NEXT and HUSH.

Subsequently, we mapped the HUSH interaction site of ZCCHC8 by generating C- and N-terminal ZCCHC8 truncations guided by conserved domains defined from structural and biochemical studies of human ZCCHC8 (Lingaraju et al., 2019; Puno and Lima, 2018) (Figures S3A and S3B). Stably integrated MYC-tagged ZCCHC8 proteins were expressed in *Zcchc8*^−/−^ cells at levels broadly comparable to the WT ZCCHC8 counterpart, and mutants containing the PRO-rich RBM7-interaction domain generally rescued RBM7 expression (Figure S3C). Conducting MPP8 IPs with the panel of ZCCHC8 proteins revealed that C-terminal truncations up to amino acid residue 249 were still able to interact with MPP8, which agreed with complementary N-terminal truncations losing the interaction (Figure 3C). The interaction domain was narrowed down to residues 154–214 (Figure 3C). However, this region overlaps with an essential surface required for MTR4 interaction (Lingaraju et al., 2019), and resultingly, ZCCHC8 mutants that lost HUSH binding were also unable to bind to MTR4 (Figures 3C and S3D). Further extensive attempts to uncouple MTR4 and MPP8 binding to ZCCHC8 proved unsuccessful (data not shown), potentially reflecting an interaction mechanism ensuring that HUSH binds to ZCCHC8 proteins associating with MTR4.

Although we were unable to separate HUSH binding to ZCCHC8 from its normal assembly with MTR4, we were able to generate HUSH-binding ZCCHC8 mutants that lost RBM7 interactions (Figures 3C and S3C). As RBM7 is required for normal NEXT function in RNA decay (Lubas et al., 2011, 2015), we could therefore address whether the RNA decay function of NEXT was required for TE RNA silencing. We analyzed RNA isolated from ZCCHC8 mutant cell lines for expression of known NEXT targets along with TE RNAs (Figure S3E). Among the ZCCHC8 fragments that support MTR4 and HUSH binding, only mutants that rescued RBM7 expression were able to support silencing of both known NEXT targets and TE RNAs (Figures S3C and S3E). We therefore conclude that the RNA decay function of ZCCHC8, and not only its HUSH connection, is required for its role in controlling TE RNA levels.

### NEXT recruitment to chromatin at HUSH-bound loci depends on MPP8

**Figure 4.**
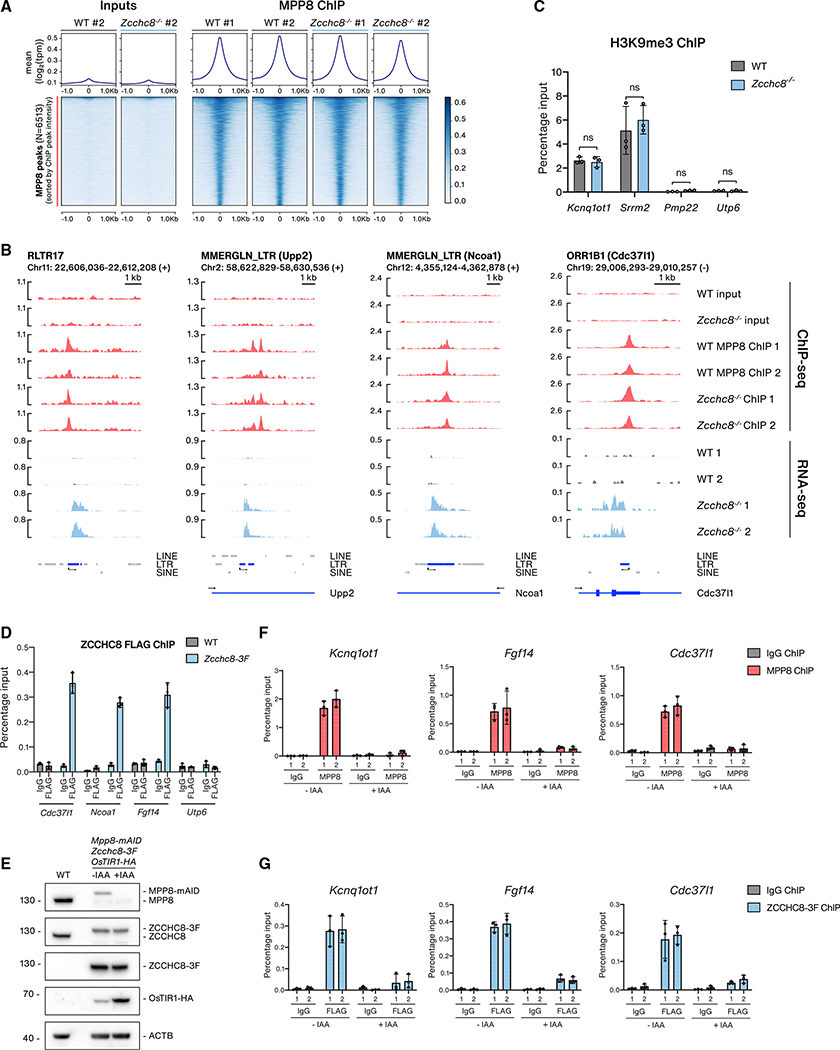
NEXT recruitment to chromatin at HUSH-bound loci depends on MPP8 (A) Metagene (upper) and heatmap (lower) profiles of signals from input and MPP8 ChIP-seq samples from WT or *Zcchc8*^−/−^ datasets within a 2 kb window centered on MPP8 peaks. MPP8 ChIP samples are from two WT replicates and two *Zcchc8*^−/−^ clones. (B) Genome browser views of four MPP8-bound loci. Displayed tracks include input and ChIP-seq data from WT or *Zcchc8*^−/−^ cells as well as stranded RNA-seq data from the same cells. Only RNA-seq data from relevant strands are displayed as in Figure 1F. For intronic TEs, the relevant host gene is included in parentheses. (C) H3K9me3 ChIP-qPCR analysis at MPP8-bound loci (*Kcnq1ot1, Srrm2*) and control regions not bound by MPP8 (*Pmp22*, *Utp6*) in WT or *Zcchc8*^−/−^ cells. Data are shown as the percentage of input with error bars indicating the SD of technical triplicates (individual data as points). Statistical significance was assessed using a two-tailed paired Student’s t test (*p < 0.05, **p < 0.01, ns, not significant). (D) qPCR analysis of IgG and FLAG ChIPs from MPP8-bound loci (*Cdc37l1*, *Ncoa1*, *Fgf14*) and a control region not bound by MPP8 (*Utp6*) in WT and *Zcchc8*-*3F* cells. Data are represented as percentage input values of three biological replicates and displayed as in (C). (E) WB analysis of lysates from WT and *Mpp8*-*mAID Zcchc8*-*3F OsTIR1*-*HA* cells either mock or IAA treated (12 h). Membranes were probed with antibodies against MPP8, ZCCHC8, FLAG, HA, and Actin (ACTB, loading control). (F) qPCR analysis of IgG and MPP8 ChIPs at MPP8-bound loci (*Kcnq1ot1*, *Fgf14*, *Cdc371l*) in from *Mpp8*-*mAID Zcchc8*-*3F OsTIR1*-*HA* samples described in (E). (G) qPCR analysis as in (F) but for IgG and FLAG ChIPs from the same samples.

To determine how NEXT and HUSH may work in concert, we next addressed whether recruitment of these complexes to TE loci might be mutually dependent. We initially took influence from mechanisms reported in *S. pombe*, where the MTREC and exosome complexes recognize nascent TE RNAs and recruit the RITS complex to trigger heterochromatin formation (Shimada et al., 2016; Verdel et al., 2004). Our previous RBM7 CLIP analyses revealed its promiscuous binding to nascent RNAPII transcripts (Lubas et al., 2015), and we reasoned that NEXT loaded onto nascent RNAs might recruit HUSH. To address this possibility, we used MPP8 ChIP-seq to compare the DNA-binding profile of MPP8 in WT and *Zcchc8*^−/−^ cells. Metagene plots displayed no notable change in MPP8-chromatin binding upon depletion of ZCCHC8 (Figure 4A), which was also evident when scrutinizing sample loci that show increased RNA levels in *Zcchc8*^−/−^ cells (Figure 4B). ChIP-qPCR experiments, using amplicons designed for either MPP8-bound (*Kcnq1ot1*, *Srrm2*) or non-bound (*Utp6*) regions, confirmed this result (Figure S4A). Furthermore, we addressed the levels of the H3K9me3 histone modification at select HUSH targets that were shown to be downregulated upon MPP8 depletion in ESCs (Müller et al., 2021). Here, we observed no significant difference between *Zcchc8*^−/−^ and WT cells (Figure 4C). Thus, we conclude that ZCCHC8 is not required for the recruitment of MPP8 to chromatin and that HUSH likely still functions in maintaining H3K9me3 levels in *Zcchc8*^−/−^ cells.

To test the reciprocal possibility that HUSH recruits NEXT to chromatin, we first asked whether NEXT would ChIP to regions bound by HUSH. Accordingly, we performed FLAG and IgG control ChIPs from WT and *Zcchc8-3F* cells. A significant enrichment of FLAG ChIP signals relative to IgG IPs was observed over MPP8-bound loci (*Cdc371l1*, *Ncoa1*, and *Fgf14*) in *Zcchc8–3F* cells, but not over a non-MPP8-bound locus (*Utp6*) (Figure 4D). ChIP analyses of the other two NEXT components, MTR4 and RBM7, using analogously generated tagged cell lines (Figure S4B), displayed similar enrichments of MTR4- and RBM7-3F signals at HUSH-bound over non-bound loci when compared to an untagged WT control cell line (Figure S4C). To distinguish whether NEXT binding to chromatin was dependent on the presence of HUSH itself, we generated *Mpp8-mAID* cell lines, harboring endogenous ZCCHC8-3F. In these cells, MPP8 was efficiently depleted following IAA treatment, with no impact on ZCCHC8-3F expression levels (Figure 4E). ChIP of MPP8 and ZCCHC8-3F was then carried out from cells, either mock or IAA treated (12 h), and qPCR analysis was performed using amplicons targeting HUSH-bound loci. Reassuringly, MPP8 ChIP signals decreased to control levels in +IAA samples at example loci (Figure 4F). Strikingly, at the same loci, ZCCHC8-3F binding was lost upon depletion of MPP8 (Figure 4G). As a control, we carried out ChIP-qPCR analysis using amplicons targeting loci that displayed NEXT sensitivity (Figures S1C and S2F) but that were not bound by HUSH (Figure S4D). ZCCHC8-3F was indeed enriched at such loci (*proRPL27a*, *proPAXIP1*, *Nanog eRNA*, *proDBNL*), but no enrichment of MPP8 was observed. Furthermore, ZCCHC8-3F binding was not affected in MPP8-depletion conditions (Figure S4D). We conclude that NEXT is recruited to chromatin at HUSH-bound loci in a HUSH-dependent manner.

### NEXT and HUSH suppress non-polyadenylated and polyadenylated TE RNAs, respectively

What might the mechanistic implications of the NEXT-HUSH connection be; do these complexes function in the same pathway or in concert to silence TE expression? One contemplative observation was that while *Zcchc8*^−/−^ ESCs are viable and maintain self-renewal properties, depletion of MPP8 is lethal in ground-state ESCs, which was shown to correlate with increased expression of L1 LINEs (Müller et al., 2021). We considered that lack of lethality of *Zcchc8*^−/−^ cells, despite their upregulated L1 transcripts, could be either due to a more essential and currently undiscovered function of MPP8 or because MPP8 and ZCCHC8 might function in different ways to dampen TE expression.

**Figure 5.**
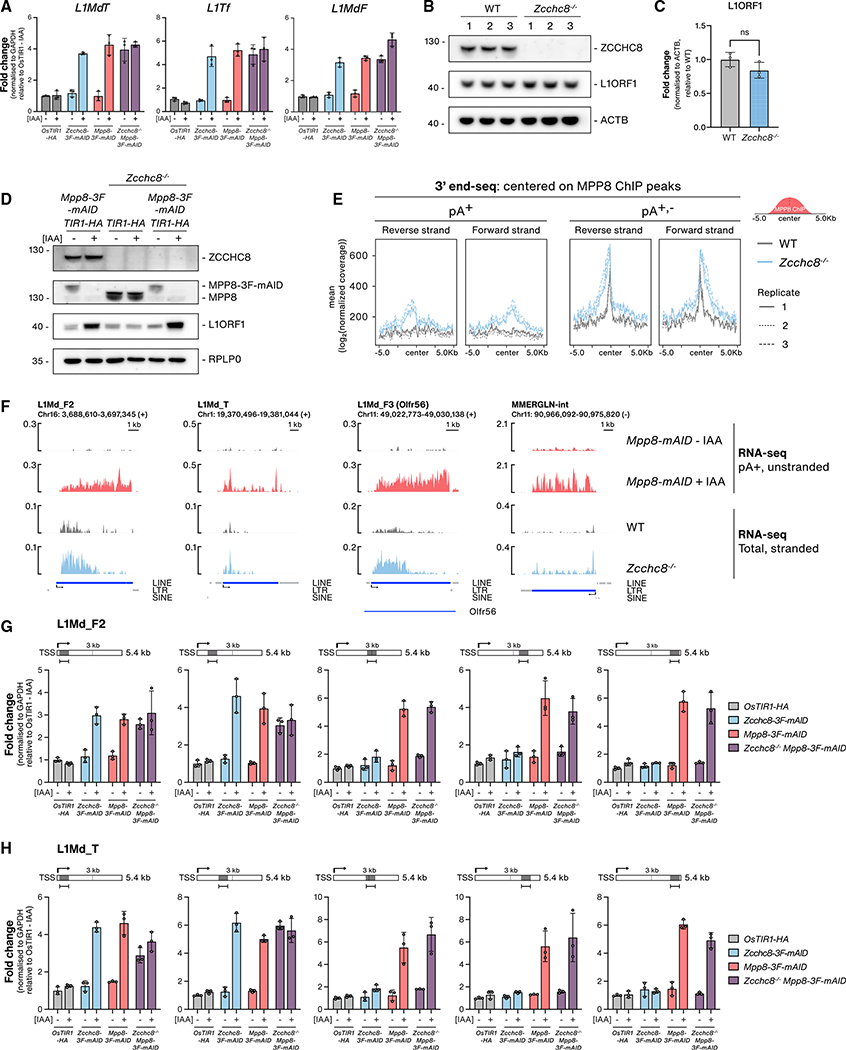
NEXT and HUSH suppress non-polyadenylated and polyadenylated TE RNAs, respectively (A) qRT-PCR analysis of L1 LINE transcripts from total RNA harvested from *OsTIR1-HA*, *Zcchc8–3F-mAID*, *Mpp8*-*3F*-*mAID*, or *Zcchc8*^−/−^ *Mpp8*-*3*F-*mAID* cell lines either mock or IAA treated (72 h). Data representation as in Figure 1H. (B) WB analysis of lysates from three biological WT replicates and three *Zcchc8*^−/−^ clonal cell lines. Membranes were probed with antibodies against ZCCHC8, L1ORF1 and Actin (ACTB, loading control). (C) Quantification of L1ORF1 protein levels from the WB in (B). Data show the average value from three replicates, normalized to ACTB levels and plotted as the fold change relative to WT samples. Statistical significance was assessed as in Figure 4C. (D) WB analysis of *Mpp8*-*3F*-*mAID*, *Zcchc8*^−/−^, and *Zcchc8*^−/−^ *Mpp8*-*3F*-*mAID* cell extracts following either mock or IAA treatment (72 h). Membranes were probed with antibodies against ZCCHC8, MPP8, L1ORF1, and RPLP0 (loading control). (E) Metagene profiles of 3′ end-seq signals from pA^+^ and pA^+,−^ 3′ end-seq libraries of WT or *Zcchc8*^−/−^ cells and displayed within a 10 kb window centered on MPP8 ChIP-seq peaks. Forward and reverse strands are plotted independently with replicates plotted separately as indicated in the legend. (F) Genome browser tracks of example upregulated L1 LINEs and LTR RNAs from RNA-seq data generated upon MPP8 or ZCCHC8 depletion. Data from *Mpp*8-*mAID* samples, either mock or IAA treated (48 h), are from pA^+^ selected, un-stranded libraries. Data from WT and *Zcchc8*^−/−^ cells are from rRNA-depleted, stranded libraries with the relevant strand data represented here. Annotations are displayed as in Figure 1F. (G) qRT-PCR analysis of L1Md_F transcripts from total RNA harvested from samples described in (A). Amplicons were designed to amplify either 5′, center, or 3′ regions of the L1Md_F2 LINE transcript as indicated in the schematics (H) As in (G) but for L1Md_T transcripts.

To examine the latter possibility, we used the mAID-tagged cell lines to compare the relative contributions of NEXT (ZCCHC8 and RBM7) and HUSH (MPP8 and TASOR) depletions on TE transcript levels, which yielded comparable LINE (*L1*-*TF*) and LTR (*MuERV*-*L*) RNA increases (Figure S5A). In agreement with previous observations (Müller et al., 2021), we did not detect any effect on B2 SINEs in HUSH depletions, demonstrating that NEXT restricts SINE expression in a HUSH-independent manner (Figure S5A). We also compared L1 transcript levels in single ZCCHC8 and MPP8 depletions versus conditional double-depletion cell lines, which revealed no noteworthy additive effects (Figure 5A). This would seemingly indicate that NEXT and HUSH operate in the same pathway. To explore this further, we assessed the extent to which L1 proteins were affected by ZCCHC8 depletion. Intact L1 RNAs encode for L1ORF1 and L1ORF2 proteins, which are required for L1 retrotransposition (Ostertag and Kazazian, 2001), and L1ORF1 levels are significantly increased upon HUSH depletions (Douse et al., 2020; Müller et al., 2021; Tunbak et al., 2020). Conspicuously, however, this was not the case in *Zcchc8*^−/−^ cells (Figures 5B and 5C), and when comparing single- and double-depletion cell lines, the increase in L1ORF1 protein levels observed upon MPP8 depletion was unaffected by co-depletion of ZCCHC8 (Figures 5D and S5B).

This prompted us to address the exact nature of ZCCHC8-sensitive TE RNAs, and we therefore generated 3′ end RNAseq libraries from total RNA isolated from WT and *Zcchc8*^−/−^ cells. For expanded focus on transcript 3′ end status, RNA samples were either mock treated or subjected to *in vitro* polyadenylation by *E. coli* polyA polymerase before carrying out polyA^+^ (pA^+^) RNA 3′ end sequencing, an approach allowing the capture of originally non-adenylated (pA^−^) transcripts and thereby generating both pA^+^ and pA^+,−^ libraries (Wu et al., 2021). Anchoring these data to MPP8 ChIP peaks demonstrated an increase in 3′ end signals downstream of peak centers upon ZCCHC8 depletion, which was most significant in the pA^+,−^ libraries (Figure 5E). Scrutinizing specific MPP8-bound loci, we further observed that ZCCHC8-sensitive TE RNAs typically harbored heterogenous 3′ ends with no distinct termination site (Figure S5C), which echoed previous observations from NEXT-targeted regions in HeLa cells (Gockert et al., 2022; Wu et al., 2020a). Likewise, transcription start site (TSS)-proximal RNA pA^+^ 3′ ends became detectable, although lowly abundant, in *Zcchc8*^−/−^ cells, mirroring the phenotype observed in NEXT-depleted HeLa cells attributed to a failsafe decay mechanism operated by PAXT (Wu et al., 2020a). We speculate that these pA^+^ 3′ ends arise from post-transcriptional polyadenylation of pA^−^ RNAs, as seen for other NEXT targets, in preparation for their targeting by PAXT (Wu et al., 2020a). This may reflect our observation of a weak HUSH-PAXT interaction (Figures 2D and 2F) but requires further characterization. We therefore surmise that the major contribution of NEXT to TE RNA regulation represents its suppression of short and pA^−^ transcripts in line with known NEXT targets.

**Figure 6.**
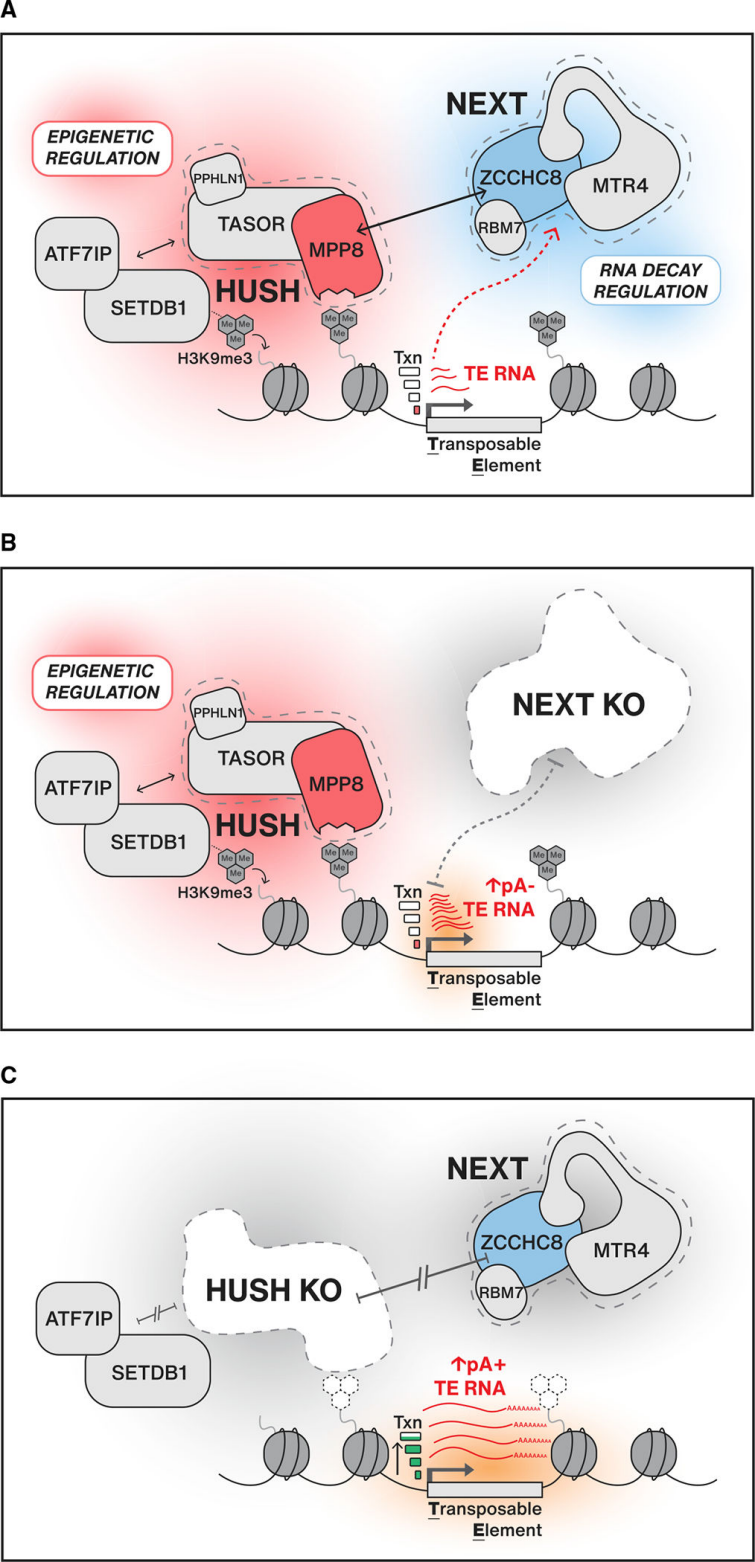
Model (A) The HUSH and NEXT complexes function to control expression of TE transcripts at either the transcriptional or post-transcriptional level, respectively. HUSH is recruited to TE loci decorated with H3K9me3 histone marks and is required for maintaining H3K9me3 levels and transcriptional (txn) suppression. NEXT is recruited to HUSH-bound loci through a physical connection that requires ZCCHC8 and MPP8. (B) In the absence of NEXT, HUSH can still bind to chromatin, regulate H3K9me3, and maintain low transcription levels. Without NEXT-mediated RNA decay, short pA^−^ transcripts from TE loci are stabilized. (C) In the absence of HUSH, H3K9me3 levels are not maintained and NEXT is no longer recruited to HUSH-bound loci. TE loci lose transcriptional repression and show an increase in full-length pA^+^ TE RNAs that, in the case of L1 LINEs, can be export competent and subsequently translated.

To relate upregulated RNA species in *Zcchc8*^−/−^ cells to their counterparts from HUSH-depletion conditions, we examined RNA-seq data obtained from *Mpp8-mAID* cells either mock or IAA treated for 48 h (Müller et al., 2021). With the caveat in mind that these data represent pA^+^ selected, unstranded libraries, example LINE and LTR loci revealed increased RNA expression across their entire transctipional units (TUs), which contrasted the TSS-proximal RNAs upregulated in *Zcchc8*^−/−^ cells (Figure 5F). Metagene analyses of LINE, LTR, and the small percentage of SINE loci bound by MPP8 presented analogous increases of TSS-proximal RNA in *Zcchc8*^−/−^ samples (Figure S5D) and similar profiles to known NEXT targets (Figure S5D, PROMPTs [Wu et al., 2020a]). To validate this observation and to address the apparent discrepancy between the two RNA-seq library preparations, we analyzed RNA from cell lines depleted of ZCCHC8 and MPP8, either individually or combined, utilizing unique qRT-PCR amplicons designed at various distances along two representative L1-LINE TUs (Figures 5G and 5H). In line with the RNA-seq results, amplicons at the 5′ ends, or within the first ~3 kb of the tested L1 TUs, displayed sensitivity to both ZCCHC8 and MPP8 depletions (Figures 5G and 5H). In contrast, amplicons positioned at TU 3′ ends only reacted to MPP8 depletion, suggesting upregulation of full-length RNA. Interestingly, the double ZCCHC8/MPP8 depletion condition phenocopied single MPP8 depletions by showing RNA upregulation across the entire TU with no additional upregulation or 5′ end positional bias. Taken together, this implies that NEXT activity toward TSS-proximal transcripts is only relevant in the presence of MPP8 and that de-repression of TE loci in HUSH-depleted conditions also “circumvents” NEXT-dependent decay, resulting in the upregulation of full-length, translation-competent LINE RNAs (Figure 6; see Discussion).

## DISCUSSION

The extent to which nuclear RNA decay systems contribute to the post-transcriptional repression of repetitive genomic elements has remained largely unexplored. The robust connection between HUSH and NEXT complexes, described here, therefore provides unprecedented insight into how a transcriptional repressor may team up with an RNA decay adaptor to efficiently reduce RNA output from TE loci in ESCs. Importantly, our data suggest that an advantage of this connection goes beyond a mere increase of NEXT binding to HUSH-controlled loci; the specific recruitment of NEXT enables targeted elimination of prematurely terminated RNAs produced as a consequence of the repressive environment originally installed by HUSH activity.

The connection between HUSH and NEXT would, at face value, resemble an analogous system in *S. pombe*, where nuclear decay and heterochromatin machineries function together to silence particular TUs, including retrotransposons. However, in the case of MTREC and RITS, their initial recruitment relies on the recognition of nascent RNAs, which are targeted by MTREC and, in turn, RITS to deposit H3K9me3 modifications (Hiriart et al., 2012; Zofall et al., 2012). Models for similar RNA-mediated recruitment of HUSH to chromatin were previously suggested but never supported by direct evidence (Douse et al., 2020; Prigozhin et al., 2020). However, it was recently shown that active transcription was required for HUSH silencing of LINE reporter constructs and that PPHLN1 was able to bind RNAs produced from HUSH target loci (Seczynska et al., 2022). While we cannot rule out an influence of RNA in HUSH recruitment, our data firmly establish that a connection to the RNA decay machinery, via NEXT, is not required (Figures 4A and 4B). Instead, we demonstrate a specific targeting of NEXT to H3K9me3 heterochromatin via HUSH (Figures 4G and 6A). Such genomic regions are often referred to as “repressive compartments”; however, it is becoming increasingly clear that they constitute a spectrum of transcriptional activities and that most heterochromatin is, to some extent, accessible to RNAPII and its termination machineries. Examples of such heterochromatic activity include transcription of siRNAs from pericentromeric regions in *S. pombe*, piRNAs from piRNA repeat clusters in *D. melanogaster*, and satellite repeat RNAs in ESCs (Andersen et al., 2017; Brennecke et al., 2007; Novo et al., 2020; Volpe et al., 2002).

It has also been shown that HUSH binds to regions of so called “leaky” heterochromatin (Liu et al., 2018; Robbez-Masson et al., 2018), and in line with this, we demonstrate that NEXT depletion triggers the appearance of short pA^−^ transcripts, terminated within the first 3 kb of HUSH-bound LINE and LTR TUs (Figures S2C, 5E–5H, and 6B). These RNAs likely arise from pervasive RNAPII transcription initiation events, as previously described for other NEXT-sensitive RNAs derived from promoter and enhancer regions (Lubas et al., 2011, 2015; Wu et al., 2020a), and we surmise that they are terminated by the Integrator (INT) complex or related transcription termination activities, which were recently shown to function genome-wide in TSS-proximal regions, generating primarily pA^−^ 3′ ends targeted by the RNA exosome (Beckedorff et al., 2020; Elrod et al., 2019; Lykke-Andersen et al., 2021). Interestingly, and with particular relevance to the present study, INT activity was found to anti-correlate with transcriptional output; lowly expressed TUs were relatively more sensitive to INT depletion (Elrod et al., 2019; Lykke-Andersen et al., 2021). Although the molecular mechanism underlying this relationship has yet to be established, it nonetheless leads us to propose that HUSH, by reducing transcription initiation levels, prepares for the action of NEXT not only by its recruitment via protein-protein interaction but also by provoking early transcription termination events. The combinatorial action of HUSH therefore enforces silencing at the transcriptional level while also promoting post-transcriptional decay of escaping RNAs through the physical connection to a nuclear RNA decay adaptor (Figure 6A). While NEXT-sensitive TE transcripts will presumably be neither mobile nor translation competent, their swift removal still provides an essential layer of quality control for the cell as such widespread and abundant RNAs, if not degraded, would soon compromise important RNA functions through the accumulation of secondary effects (Garland and Jensen, 2020; Schmid and Jensen, 2018).

In the absence of HUSH, TE loci show reduced H3K9me3 levels and loss of transcriptional suppression (Liu et al., 2018; Müller et al., 2021; Tchasovnikarova et al., 2015). We suggest that such increased transcriptional output tip the scales from a predominant TSS-proximal termination toward productive elongation, leading to transcript processing and termination by the canonical cleavage and polyadenylation machinery (Figure 6C). This distinction between NEXT and HUSH depletions is reflected by our observation that L1ORF1 protein levels are increased only upon removal of MPP8 but not of ZCCHC8 (Figures 5B–5D). We also do not observe additive effects on LINE RNA levels upon co-depletion of ZCCHC8 and MPP8, which at first glance seems paradoxical given that both complexes aim to silence different transcripts generated from the same loci (Figure 5A). However, in the described scenario, enhanced transcription elongation in HUSH-depletion conditions will negate the production of NEXT substrates, rationalizing the resemblance of double ZCCHC8/MPP8 and single MPP8 depletion phenotypes (Figures 5G and 5H). Given the relatively restricted action of HUSH at RNAPII transcribed TEs, we speculate that this molecular principle may not be exclusive to the NEXT-HUSH connection but also may underlie other yet unidentified connections between epigenetic regulators and post-transcriptional activities. In particular, RNAPIII-transcribed SINEs constitute a large proportion of NEXT-sensitive TEs yet remain impervious to HUSH depletions (Figure S5A) (Müller et al., 2021), suggesting an alternative, currently uncharacterized, recruitment mechanism.

### Limitations of the study

The repetitive nature of TEs requires specialized approaches to tackle issues of mapping alignments and primer design for qRT-PCR analyses. Compromises must be taken depending on the questions asked. We initially used a multi-mapping alignment approach for read count-based DE analyses between samples (Figure 1). While this allowed a general overview of upregulated TEs between conditions, uniquely mapped reads were required when interrogating individual loci. However, while unique reads provide good coverage of evolutionary older and diverged TE families, they generally perform worse for younger, less polymorphic TE families. Nevertheless, we observed similar trends of upregulated TEs at the family level when using either mapping approach (Figures 1D and S1F). For low-throughput analyses of TEs using qRT-PCR, amplicons can be designed for TE RNAs at either a family (e.g., *L1*Tf) or a locus level. For the latter, we designed amplicons so that at least one primer was specific to the TE of interest.

Our study utilized genomic KOs of ZCCHC8 and ZFC3H1 generated by CRISPR-Cas9. This allowed for a complete loss-of-function cellular condition but might also yield indirect effects or compensatory changes due to long-term depletions. To remedy this, we complemented our analyses utilizing degron-based rapid depletion systems with a reduced risk of secondary effects.

Finally, we conducted our study with mouse ESCs, displaying lower levels of DNA methylation than their somatic cell counterparts and with a possible stronger reliance on alternative mechanisms to regulate TEs. Therefore, how HUSH and NEXT may function to silence TEs in differentiated cells, with more dominant DNA methylation mechanisms, remains an open question.

## STAR★METHODS

### RESOURCE AVAILABILITY

#### Lead contact

Further information and requests for resources and reagents should be directed to and will be fulfilled by the lead contact, Torben Heick Jensen (thj@mbg.au.dk).

#### Materials availability

All unique/stable reagents generated in this study are available from the Lead Contact without restriction.

#### Data and code availability

- All RNA-seq, 3′ end-seq and ChIP-seq datasets generated during this study are available at the Gene Expression Omnibus (GEO) under accession code GSE178550. Raw image files are deposited on Mendeley Data and are available at https://doi.org/10.17632/kxmdksmt9c.1.
- This paper does not report original code.
- Any additional information required to reanalyse the data reported in this paper is available from the lead contact upon request.

### EXPERIMENTAL MODEL AND SUBJECT DETAILS

#### mES cell culture and transfections

E14TG2a mouse ESCs (male genotype, XY) and descended cell lines were cultured on 0.2% gelatin coated plates in 2i/LIF containing medium (1:1 mix of Neurobasal (GIBCO) and DMEM/F12 (GIBCO) supplemented with 1× Pen-Strep (GIBCO), 2 μM Glutamax, 50 μM β-mercaptoethanol (GIBCO), 0.1 mM Non-Essential Amino Acids (GIBCO), 1 mM Sodium Pyruvate (GIBCO), 0.5× N2 supplement (GIBCO), 0.5× B27 supplement (GIBCO), 3 mM GSK3i (CHIR99021), 1 mM MEKi (PD0325901) and Leukemia Inhibitory Factor (LIF, produced in house)) at 37°C, 5% CO_2_. Cells were passaged every 2–3 days by aspirating medium, dissociating cells with 0.05% Trypsin-EDTA (GIBCO) briefly at 37°C before the addition of an equal volume of 1× Typsin Inhibitor (Sigma) and gentle disruption by pipetting. Cells were pelleted by centrifugation to remove trypsin before resuspending and plating at ~8×10^4^ cells/mL. Cell lines were transfected with single plasmids using Viafect (Promega) or multiple plasmids using Lipofectamine 3000 (Thermo) in 6 well plates. For antibiotic selection, Blasticidin (BSD) was used at 10 μg/mL, Hygromycin B was used at 100 μg/mL, Genetecin (G418) was used at 250 μg/mL, Puromycin was used at 1 μg/mL. For depletion in AID-tagged cell lines, 750 μM Indole-3-acetic acid sodium salt (IAA, Sigma) was supplemented to the grown medium and cells were incubated for indicated time points before harvest. A full list of cell lines used or generated in this study is found in the key resources table.

### METHOD DETAILS

#### CRISPR-Cas9 knockout cells

Generation of *Zfc3h1*^−/−^ KO ES cell lines was described in Garland et al. (2019). *Zcchc8*^−/−^ KO cell lines were generated in a similar way. Single guide (sg) RNAs targeting the first exon (Table S1) were designed using the CHOPCHOP tool (v3) (Labun et al., 2019) and cloned into the pSLCas(BB)-2A-GFP vector (pX458, Addgene plasmid ID: 48138) as previously described (Ran et al., 2013). sgRNA plasmids were transfected into ES cells using Viafect (Promega). Single cell clones were isolated by GFP sorting using FACS into 0.2% gelatin coated 96 well plates containing 2i/LIF media and were subsequently expanded. Clonal cell lines were screened by western blotting (WB) analysis and genotyped by sanger sequencing of amplified genomic DNA around the sgRNA target site. Three independent *Zcchc8*^−/−^ cell lines were derived from expanded single cell clones.

#### CRISPR-Cas9 knock-in cells

CRISPR-Cas9 mediated genomic knock-ins of C-terminal 3xFLAG (3F) or 3xFLAG-mini-AID (3F-mAID) tags were carried out using pGolden (pGCT) homology dependent repair (HDR) donor vectors. Plasmids were generated containing gene specific 5′ and 3′ homology arms (~500 bp) amplified from WT ES cell genomic DNA and cloned into pGCT donor vectors. Epitope tagging donor plasmids comprised of a 5′ homology arm – [3xFLAG] – P2A – [HYG/NEO/BLAST/PURO] – 3′ homology arm. AID tagging donor plasmids comprised of a 5′ homology arm – [3xFLAG] – mAID – P2A – [HYG/NEO/BLAST/PURO – 3′ homology arm]. For a full list of HDR donor plasmids used, see the key resources table. sgRNAs targeting the 3′ UTR of genomic loci were cloned into pSLCas(BB)-2A-PURO vectors (pX459 Addgene plasmid ID: #48139) as described above (Table S1). Cells were co-transfected using Lipofectamine 3000 (Thermo) with 2 donor plasmids harboring distinct selection markers along with a sgRNA/Cas9 vector in a 1:1:1 ratio. 3F-tagging was carried out in WT ES cells and 3F-mAID-tagging was carried out in *OsTIR1*-*HA* expressing ES cells. Colonies were maintained under double selection for the donor plasmid markers to increase the likelihood of homozygous knock-in clones. Single cell clones, that survived the selection process, were expanded and screened by western blotting analysis and confirmed by sanger sequencing of the targeted locus.

#### cDNA cloning and exogenous expression of ZCCHC8

ZCCHC8 cDNA constructs were cloned, using a full-length cDNA plasmid as a template (pUC19[mZCCHC8], Sino Biological), into a piggyBAC (pBAC) vector containing an N-terminal MYC tag and BSD selection marker using NEBuilder HiFi DNA assembly (NEB). Generated constructs are listed in the key resources table. *Zcchc8*^−/−^ mES cells were transfected with pB-MYC-ZCCHC8*-BSD vectors along with a piggyBAC transposase expressing vector (pBase) in a 1:1 ratio using Viafect (Promega). Cell pools were selected with BSD for ~7–10 days or until negative control cells no longer survived. Expression of constructs were validated by western blotting analysis using MYC antibodies.

#### RNA isolation and RTqPCR analysis

Total RNA was isolated using the RNeasy Mini Kit (QIAGEN), according to the manufacturer’s instructions, or by Trizol extraction (Thermo) using the standard protocol. Extracted RNA was treated with TURBO DNase (Invitrogen) following the manufacturer’s instructions followed by cDNA preparation from 1 μg RNA using SuperScript III reverse transcriptase (Invitrogen) and a mix of 80 pmol random primers and 20 pmol dT20 primers. qPCR was performed using Platinum SYBR Green using an AriaMx Real-Time PCR machine (Agilent Technologies). Primers for RTqPCR are listed in Table S2. To detect TE transcripts, primers were either designed to detect RNAs at the generic family level or for specific loci. For generic targets, primers were designed to amplify regions common between classes of TEs. For unique, locus-specific targets, amplicons were designed so that at least one primer, ideally both, was specific to the TE locus of interest.

#### RNA-seq library preparation

Standard RNA-seq data from triplicate WT and *Zfc3h1*^−/−^ samples were described previously (Garland et al., 2019) (GEO: GSE137491) and RNA from *Zcchc8*^−/−^ clones were sequenced in the same batch. RNA-seq libraries were prepared from 1 μg of total RNA using the TruSeq Stranded RNA library prep kit with RiboZero Gold (Illumina), according to the manufacturer’s instructions. Three biological replicates from each sample were prepared. RNA integrity and library quality were assessed on a Bioanalyzer 2000 using RNA Nano and DNA 1000 chips (Agilent), respectively. Libraries were quantified and normalized for multiplexing using the KAPA library quantification Kit for Illumina (Kapa Biosystems) and a Qubit Flourometer (Thermo) before sequencing on an Illumina NextSeq 550 (75-bp, paired end). RNA-seq data from MPP8-depletion conditions was described in (Müller et al., 2021) (GEO: GSE150926).

For RNA 3′ end sequencing samples, 2 μg of TURBO DNase treated total RNA was either mock treated or incubated with *E.coli* poly(A) polymerase (EPAP, Invitrogen) at 3°C for 30 min in 1 X reaction buffer, 2.5 mM MnCl_2_, +/− 0.4 U EPAP, 0.8 U Ribolock RNase inhibitor (Thermo) and 1 mM ATP. RNA was purified using PureLink RNA Micro purification kits (Ambion) following the manufacturer’s instructions. rRNA depletion was carried out using RiboCop rRNA Depletion Kit (Lexogen) according to the manufacturer’s instructions. RNA 3′ end-seq was carried out using the QuantSeq 3′ mRNA-seq FW + UMI strategy (Lexogen GmbH). Libraries were prepared by Lexogen and this included the addition of spike-ins ERCC Mix1 and SIRV E0. Illumina single read 75 nucleotides sequencing of those libraries was carried out by Lexogen. A single library was sequenced for each treatment of RNA from the 3-independent control and *Zcchc8*^−/−^ cell lines.

#### Processing and analysis of RNA-seq data

For standard RNA-seq, raw reads were trimmed using Trim Galore (v0.4.4, https://www.bioinformatics.babraham.ac.uk/projects/trim_galore/) to remove Illumina adaptors, low-quality bases with Phred score lower than 20, and reads shorter than 25 bp; reads were further trimmed using parameters–clip_R1 13–clip_R2 13–three_prime_clip_R1 1–three_prime_clip_R2 1. Trimmed reads were mapped to mouse genome mm10 using STAR (v2.7.3a) (Dobin et al., 2013) in which reads with maximum multiple alignment of no more than 100 were kept using parameters–winAnchorMultimapNmax 100–outFilterMultimapNmax 100. Mapped reads, overlapping with genes and TEs, were counted using TElocal (v0.1.0), which can quantify TEs at the locus level by using both uniquely mapped and multi-mapped reads. For genes, GENCODE M22 annotation was used; and for TEs, the pre-built annotation provided by TElocal was used. Differential expression analysis was performed using edgeR (McCarthy et al., 2012; Robinson et al., 2010) with default parameters.

Genomic location of TEs was annotated based on a hierarchical ranked classification as previously described (Wu et al., 2020b). GENCODE M22 annotation was used and the categories in priority order were as follows: promoter – within ± 100 bp of annotated TSSs of genes; fiveUTR – 5′ UTRs of transcripts with annotated coding regions (CDS); threeUTR – 3′ UTRs of transcripts with annotated CDS; CDS – annotated CDS; exon – annotated exons; intron – annotated introns; antisense – antisense strand of annotated transcripts; intergenic – regions not overlapping with any of the previous genomic categories. To generate visualization, the strand specific genomic coverage was calculated and normalized to library size using bamCoverage from deepTools (v2.5.3) (Ramírez et al., 2014).

RNA 3′ end-seq data provided by Lexogen were already split by barcodes and UMI information was appended to read names, which were then quality controlled and trimmed using the bbduk.sh script from the BBMap software (v37.62, https://sourceforge.net/projects/bbmap/) with parameters ktrim = r useshortkmers = t mink = 5 qtrim = r trimq = 10 minlength = 20 and providing Illumina sequencing adapters as reference file. A-tails longer than 4 nucleotides on the 3′ end of cleaned reads were trimmed off using a custom awk script and only reads with more than 20 nucleotides after this step were used for subsequent mapping. Here, a custom index, concatenating mouse genome mm10 and sequences for Lexogen ERCC and SIRV spike ins (SIRV-Set 3, Lexogen GmbH), was prepared. This merged genome was indexed using STAR (v.2.7.3a) (Dobin et al., 2013) with settings ‘–runMode genomeGenerate–genomeSAindexNbases 11’ and otherwise default settings (i.e., providing no splice-junction information). Reads were then mapped to this index using the STAR aligner (v2.7.3a) (Dobin et al., 2013)’ together with SAMtools (v.1.9) (Li et al., 2009) with settings ‘–outFilterType BySJout–outFilterMultimapNmax 1’ for single mapping reads and ‘–outFilterType BySJout– outFilterMultimapNmax 1000’ to maintain also multimapping reads. Duplicate reads were removed via the included UMI information, using the dedup function from UMI-tools (v1.0.1) (Smith et al., 2017). BAM files were indexed using SAMtools and coverage of single mapping reads within last exons of RefSeq annotations were obtained using the multicov function from BEDtools (v2.29.2) in strand-specific mode. Normalization factors were obtained from those last exon counts using the estimateSizeFactors function from R package DESeq2 (v1.28.0) (Love et al., 2014), with at least a total of 100 reads summed over all libraries. Size factors were checked to be generally correlated with spike-in derived read numbers without indications for KO or EPAP treatment-specific biases. Normalized coverage tracks were then produced using the genomecov function from bedtools with parameters ‘-bg –3 -scale 1/sizefactor’ and ‘-strand +’ or ‘-strand -’ for plus and minus strand coverage, respectively. Coverage for major mouse chromosomes was sorted using bedtools sort, the average of the 3 replicates computed and converted to bigwig format using bedGraphToBigwig function from UCSC tools (v357) (Kent et al., 2010).

#### Chromatin immunoprecipitation (ChIP)

Cells were crosslinked with 1% formaldehyde (Sigma) for 10 min before quenching with 0.25 M glycine and washed in PBS. Cells were harvested in SDS buffer (50 mM Tris pH 8.0, 100 mM NaCl, 5 mM EDTA pH 8.0, 0.5% SDS, 0.02% v/v NaN_3_) supplemented with protease inhibitors. Nuclei were pelleted by centrifugation at 400 rcf for 15 min and resuspended in IP buffer (2x volumes SDS buffer and 1 volume Triton dilution buffer (100 mM Tris pH 8.0, 100 mM NaCl, 5 mM EDTA pH 8.0, 5% v/v Triton X-100, 0.02% v/v NaN_3_). Samples were sonicated to shear chromatin to an average size of 200 bp using either a Bioruptor (Diagenode) or Covaris S2 sonicator. Chromatin IPs were performed using antibodies as indicated (See key resources table), overnight at 4°C with Protein G Dynabeads (Thermo). Beads were subsequently washed 3 times in low salt wash buffer (50 mM HEPEs pH 7.5, 150 mM NaCl, 1% v/v Triton X-100, 1 mM EDTA pH 8.0, 0.1% Sodium deoxycholate, 0.02% v/v NaN_3_), 2 times in high salt wash buffer (50 mM HEPEs pH 7.5, 500 mM NaCl, 1% v/v Triton X-100, 1 mM EDTA pH 8.0, 0.1% Sodium deoxycholate, 0.02% v/v NaN_3_) and once in IP buffer. Beads were eluted and de-crosslinked overnight in elution buffer (100 mM NaHCO_3_, 1% SDS) with RNaseA at 65°C shaking at 1000 rpm. Samples were treated with proteinase K at 60°C for 1 h before purifying DNA using PCR purification kit (QIAGEN, Thermo). ChIP samples were either analyzed by qPCR, using primers listed in Table S3, or used to generate libraries using the NEBNext Ultra II Kit (NEB) according to the manufacturer’s instructions. Libraries were sequenced by Illumina NextSeq 550 (75 bp, paired end).

#### Processing and analysis of ChIP-seq data

MPP8 ChIP-seq was performed in two batches, with each comprising one independent biological replicate of WT and *Zcchc8*^−/−^ cells. Input samples were sequenced for WT of batch 1 and both WT and *Zcchc8*^−/−^ for batch 2. Raw reads were trimmed as described for RNA-seq data above. Trimmed reads were mapped to mouse genome mm10, using bowtie2 with default settings, in which only best alignment is reported for multiple alignments. MPP8 peaks were called using MACS2 (v2.1.1) (Zhang et al., 2008) with parameters–qvalue 0.05–broad–broad-cutoff 0.3. ENCODE blacklisted peaks (Dunham et al., 2012) and low quality peaks (−log10(qvalue) ≤ 1.5) were filtered out. For each batch, a MPP8 reference peak set was obtained by pooling MPP8 peaks of WT and *Zcchc8*^−/−^ and merging overlapping peak regions into a single region using mergeBed from bedtools (v2.26.0) (Quinlan and Hall, 2010). Final MPP8 peak annotations were derived by intersection of the peak sets from the 2 batches using intersectBed from bedtools. For visualization, the genomic coverage was calculated and normalized to library size using bamCoverage from deepTools (v2.5.3) (Ramírez et al., 2014).

#### Western blotting analysis

Whole cell protein lysates were prepared using either RSB100 (10 mM Tris-HCl pH 7.5, 100 mM NaCl, 2.5 mM MgCl_2_, 0.5% NP-40, 0.5% Triton X-100) or TOPEX+ buffer (Riising et al., 2014) (50 mM Tris-HCl pH 7.5, 300 mM NaCl, 0.5% Triton X-100, 1% SDS, 33.3 U/mL Benzonase) freshly supplemented with protease inhibitors (Roche). Samples were denatured by the addition of NuPAGE Loading Buffer (Invitrogen) and NuPAGE Sample Reducing Agent (Invitrogen) before boiling at 95°C for 10 min. SDS-PAGE was carried out on either NuPAGE 4%–12% Bis-Tris or 3%–8% Tris-Acetate gels (Invitrogen). Western blotting analysis was carried out according to standard protocols with the antibodies listed in the key resources table and HRP conjugated secondary antibodies (Agilent) or Veriblot (Abcam). Bands were visualized by Super Signal West Fempto chemiluminescent ECL (Thermo) and captured using an Amersham Imager 600 or ImageQuant 800 imaging systems (GE Healthcare). Images were processed and quantified using ImageJ (v1.51) (Schneider et al., 2012).

#### IP experiments

For whole cell extract IPs, 2×10^7^ cells/IP were standardly extracted in HT150 extraction buffer (20 mM HEPES pH 7.4, 150 mM NaCl, 0.5% v/v Triton X-100) freshly supplemented with protease inhibitors. Lysates were sheared by sonication (3 × 5 s, amplitude 2) and cleared by centrifugation at 18,000 rcf for 20 min. Clarified lysates were incubated with primary antibodies (see key resources table) overnight at 4°C with Protein G Dynabeads (Thermo). Beads were washed 3 times with HT150 extraction buffer, transferring beads to a fresh tube on the final wash. Proteins were eluted by boiling in 1X NuPAGE loading buffer (Invitrogen) for 5 min. Supernatants were mixed with 10X Reducing Agent (Invitrogen) and denatured for a further 5 min at 95°C before proceeding with western blotting analysis. For IP stringency tests, samples were extracted in a range of HT buffers (20 mM HEPES pH 7.4, 0.5% v/v Triton X-100) with varying NaCl concentrations (100 mM, 200 mM, 300 mM, 500 mM, 1M). Following incubation with antibodies and Protein G Dynabeads, samples were washed with corresponding extraction buffer and proceed as described above. For benzonase treated IPs, samples were extracted and washed in HTM200 buffer (20 mM HEPES pH 7.4, 200 mM NaCl, 1 mM MgCl_2_, 0.5% Triton X-100). After the final washing step, beads were resuspended in HTM200 buffer and either mock or treated with ~250 units Benzonase (Sigma) at 25°C for 20 min in a thermomixer at 1000 rpm. Beads were pelleted and supernatants were collected for Benzonase elution samples. The beads were washed 2x in HTM200 before elution of bound proteins using the standard protocol described above. For IPs from chromatin samples, ~8×10^6^ cells were subjected to subcellular fractionation to remove cytoplasmic and soluble nucleoplasmic proteins and was adapted from (Conrad and Ørom, 2017). Cell pellets were resuspended in nuclear isolation buffer (NIB) (10 mM Tris pH 7.4, 150 mM NaCl, 0.15% NP-40) supplemented with protease inhibitors and lysed at 4°C on a rotating wheel for 5 min. Lysates were overlaid onto 1 mL Sucrose buffer (10 mM Tris pH 7.4, 150 mM NaCl, 24% w/v sucrose) in a LoBind tube (Eppendorf). Nuclei were pelleted at 1000 × rcf for 10 min, 4°C and washed with PBS-EDTA (1× PBS, 500 mM EDTA). Nuclei were resuspended in glycerol buffer (20 mM Tris pH 7.4, 75 mM NaCl, 0.5 mM EDTA, 50% v/v glycerol) before quickly lysing in nuclear lysis buffer (NLB) (10 mM Tris pH 7.4, 300 mM NaCl, 7.5 mM MgCl_2_, 0.2 mM EDTA, 1 M urea, 1% NP-40). Insoluble material, containing chromatin was pelleted at 18,000 rcf for 2 min at 4°C and washed 2x in PBS-EDTA, 2x in PBS supplemented with protease inhibitors. Chromatin pellets were resuspended in HTM200 + 2.5 mM CaCl_2_ + 300 units micrococcal nuclease (MNase, Sigma) and incubated at 37°C for 10 min at 1000 rpm. Samples were subsequently treated as lysates and subjected to sonication and clarification as above before use in IP experiments. Beads were washed in HTM200 before standard elution of proteins as described above.

#### Glycerol gradient ultracentrifugation

Glycerol gradient sedimentation analyses were performed as described in Garland et al. (2019) with some modifications. Harvested cells (~2×10^8^) were resuspended in BC100 buffer (Chu et al., 2014) (5 mM HEPES pH 7.5, 100 mM NaCl, 1mM MgCl_2_, 0.5 mM EGTA, 0.1 mM EDTA, 10% v/v glycerol, 1 mM DTT) supplemented with protease inhibitors, lysed by sonication (3 × 5 s, amplitude 2) and centrifuged at 18,000 rcf for 20 min. Clarified extracts were overlaid onto 10%–50% glycerol gradients prepared in BC100 buffer and centrifuged at 35,000 rpm for 24 h using a SW41 rotor (Beckman). Gradients were separated into 18 fractions and samples were prepared for SDS-PAGE and analyzed by western blotting analysis. Input samples were also retained to run in parallel with the fractions.

#### Data visualization

Plotting of genome browser tracks was generated in Python 3 using the SparK plotting tool (v.2.6.2) (Kurtenbach and William Harbour, 2019) using BedGraph files. Metagene heatmaps and profiles were produced using computeMatrix and plotHeatmap functions from the deepTools software suite (v3.4.3) (Ramírez et al., 2014) combined with custom python scripts for log_2_ transformation and display (Wu et al., 2020a). Heatmaps and profiles for ChIP data were prepared for regions ± 1kb from ChIP peak center, using 50bp bins and peaks sorted using total ChIP signal per ± 1kb from the center. For plotting, a pseudocount of 1 was added to all bins and values were log_2_ transformed. Heatmaps and profiles for RNA-seq and 3′ end-seq data were prepared using normalized signals of replicate average value for regions ± 5kb from peak centers using 100bp bins and regions sorted using the ChIP signal as above. Again, for plotting, a pseudocount of 1 was added to all bins and values then log_2_ transformed. RTqPCR and qPCR data was imported from the AriaMx software (v1.71, Agilent) and plotted using Graphpad Prism 9.0.0. Heatmap data to depict evolutionary conserved amino acids was generated using data from the Aminode webtool (Chang et al., 2018) and plotted in Graphpad Prism 9.0.0.

### QUANTIFICATION AND STATISTICAL ANALYSIS

All statistical analyses are indicated in the legends to the relevant figure.

## Supplementary Material

1

## Figures and Tables

**KEY RESOURCES TABLE:** 

REAGENT or RESOURCE Antibodies	SOURCE	IDENTIFIER

Mouse monoclonal anti-ACTIN	Sigma-Aldrich	Cat# A2228; RRID:AB_476697
Rabbit polyclonal anti-DIS3	Sigma-Aldrich	Cat# HPA039281; RRID:AB_10795583
Rabbit polyclonal anti-EXOSC4 (RRP41)	Brouwer et al., 2001	N/A
Mouse monoclonal anti-FLAG M2	Sigma-Aldrich	Cat# F1804; RRID:AB_262044
Rabbit polyclonal anti-H3	Abcam	Cat# ab1791; RRID:AB_302613
Rabbit polyclonal anti-H3K9me3	Abcam	Cat# ab8898; RRID:AB_306848
Rat monoclonal anti-HA	Sigma-Aldrich	Cat# 11867423001; RRID:AB_390918
Rabbit polyclonal IgG	Cell Signaling	Cat# 2729; RRID:AB_1031062
Rabbit monoclonal anti-L1ORF1	Abcam	Cat# ab216324
Rabbit polyclonal anti-MPP8	Proteintech	Cat# 16796-1AP; RRID:AB_2266644
Rabbit polyclonal anti-MTR4 (SKIV2L2)	Abcam	Cat# ab70551; RRID:AB_1270701
Rabbit monoclonal anti-MYC	Cell Signaling	Cat# 2278; RRID:AB_490778
Mouse monoclonal anti-MYC	Abcam	Cat# ab32; RRID:AB_303599
Rabbit monoclonal anti-PABPN1	Abcam	Cat# ab75855; RRID:AB_1310538
Rabbit polyclonal anti-PPHLN1	Fisher	Cat# BS7872R
Rabbit polyclonal anti-RBM7	Sigma-Aldrich	Cat# HPA013993; RRID:AB_1856137
Rabbit monoclonal anti-RPLP0	Abcam	Cat# ab192866; RRID:AB_2814809
Rabbit polyclonal anti-TASOR (FAM208a)	Thermo Fisher	Cat# PA5-89059; RRID:AB_2805327
Rabbit polyclonal anti-ALPHA-TUBULIN	Rockland	Cat# 600-401-880; RRID:AB_2612816
Mouse monoclonal anti-VINCULIN (VCL)	Sigma-Aldrich	Cat# V9131; RRID:AB_477629
Rabbit polyclonal anti-ZC3H18	Sigma-Aldrich	Cat# HPA040847; RRID:AB_10794865
Mouse polyclonal anti-ZCCHC8	Abcam	Cat# ab68739; RRID:AB_1271512
Rabbit polyclonal anti-ZFC3H1	Sigma-Aldrich	Cat# HPA007151; RRID:AB_1846133

Chemicals, peptides, and recombinant proteins		

GSK3 inhibitor (CHIR99021)	Sigma-Aldrich	Cat# SML1046
MEK1/2 inhibitor (PD0325901)	Sigma-Aldrich	Cat# PZ0162
Indole-3-acetic acid sodium salt (IAA)	Sigma-Aldrich	Cat# I5148-10G
Trizol	Thermo Fisher	Cat# 15596026

Critical commercial assays		

RNeasy Mini Kit	QIAGEN	Cat# 74104
TURBO DNase kit	Thermo Fisher	Cat# AM2238
SuperScript III Reverse Transcriptase	Thermo Fisher	Cat# 1808044
Platinum SYBR Green qPCR SuperMix	Thermo Fisher	Cat# 11733046
Ribolock RNase Inhibitor	Thermo Fisher	Cat# EO0381
TruSeq Stranded Total RNA Library Prep Kit with RiboZero Gold	Illumina	Cat# 20020598
KAPA Library Quantification Kit for Illumina	KAPA Biosystems	Cat# KK4824
NEBNext Ultra II DNA Library Prep Kit	NEB	Cat# E7645S
RiboCop rRNA Depletion kit	Lexogen GmbH	Cat# 037.96
*E. coli* poly(A) polymerase (E-PAP)	Thermo Fisher	Cat# AM2030
Protein G Dynabeads	Thermo Fisher	Cat# 10009D
Lipofectamine 3000 Transfection Reagent	Thermo Fisher	Cat# L300001
Viafect Transfection Reagent	Promega	Cat# E4981
Benzonase nuclease	Millipore	Cat# 70746
Micrococcal Nuclease (MNase)	Sigma-Aldrich	Cat# N3755
RNaseA	Sigma-Aldrich	Cat# R6148
Proteinase K	Thermo Fisher	Cat# EO0491
NEBuilder HiFi DNA Assembly cloning kit	NEB	Cat# E5520S
GeneJET PCR Purification Kit	Thermo Fisher	Cat# K0701

Deposited data		

Raw and analyzed data (RNA-seq, ChIP-seq and RNA 3′ end-seq)	This study	GEO: GSE178550
WT and *Zfc3h1^−/−^* RNA-seq	Garland et al., 2019	GEO: GSE137491
MPP8mAID RNA-seq	Müller et al., 2021	GEO: GSE150926

Experimental models: Cell lines		

Mouse ES-E14TG2a	ATCC	Cat# CRL-1821
Mouse ES-E14TG2a *Zfc3h1^−/−^* #1	Garland et al., 2019	N/A
Mouse ES-E14TG2a *Zfc3h1^−/−^* #2	Garland et al., 2019	N/A
Mouse ES-E14TG2a *Zfc3h1^−/−^* #3	Garland et al., 2019	N/A
Mouse ES-E14TG2a *Zcchc8^−/−^* #1	This study	N/A
Mouse ES-E14TG2a *Zcchc8^−/−^* #2	This study	N/A
Mouse ES-E14TG2a *Zcchc8^−/−^* #3	This study	N/A
Mouse ES-E14TG2a *OsTIR1-HA*	This study	N/A
Mouse ES-E14TG2a *OsTIR1-HA Zcchc8-3F-mAID*	This study	N/A
Mouse ES-E14TG2a *OsTIR1-HA Rbm7-3F-mAID*	This study	N/A
Mouse ES-E14TG2a *OsTIR1-HA Zfc3h1-3F-mAID*	This study	N/A
Mouse ES-E14TG2a *OsTIR1-HA Mpp8-3F-mAID*	This study	N/A
Mouse ES-E14TG2a *OsTIR1-HA Tasor-3F-mAID*	This study	N/A
Mouse ES-E14TG2a *OsTIR1-HA Zcchc8^−/−^ Mpp8-3F-mAID*	This study	N/A
Mouse ES-E14TG2a TET::*OsTIR1-FLAG Mtr4-3F-mAID*	This study	N/A
Mouse ES-E14TG2a *Mpp8-mAID*	Müller et al., 2021	N/A
Mouse ES-E14TG2a *Mpp8-mAID OsTIR1-HA Zcchc8-3F*	This study	N/A
Mouse ES-E14TG2a *Mtr4-3F*	This study	N/A
Mouse ES-E14TG2a *Zcchc8-3F*	This study	N/A
Mouse ES-E14TG2a *Rbm7-3F*	This study	N/A
Mouse ES-E14TG2a *Tasor-3F*	This study	N/A
Mouse ES-E14TG2a *Zcchc8^−/−^ MYC-Zcchc8^1–709^*	This study	N/A
Mouse ES-E14TG2a *Zcchc8^−/−^ MYC-Zcchc8^1–660^*	This study	N/A
Mouse ES-E14TG2a *Zcchc8^−/−^ MYC-Zcchc8^1–337^*	This study	N/A
Mouse ES-E14TG2a *Zcchc8^−/−^ MYC-Zcchc8^1–249^*	This study	N/A
Mouse ES-E14TG2a *Zcchc8^−/−^ MYC-Zcchc8^1–153^*	This study	N/A
Mouse ES-E14TG2a *Zcchc8^−/−^ MYC-Zcchc8^1–83^*	This study	N/A
Mouse ES-E14TG2a *Zcchc8^−/−^ MYC-Zcchc8^214–709^*	This study	N/A
Mouse ES-E14TG2a *Zcchc8^−/−^ MYC-Zcchc8^250–709^*	This study	N/A
Mouse ES-E14TG2a *Zcchc8^−/−^ MYC-Zcchc8^288–709^*	This study	N/A

Oligonucleotides		

sgRNA oligonucleotides	See Table S1	N/A
qRT-PCR oligonucleotides	See Table S2	N/A
ChIP-qPCR oligonucleotides	See Table S3	N/A

Recombinant DNA		

pBAC[OsTIR1-HA] ZEO	This study	N/A
pBAC[OsTIR1-FLAG] BLAST	This study	N/A
pGCT[ZCCHC8-3F-mAID] HYG	This study	N/A
pGCT[ZCCHC8-3F-mAID] NEO	This study	N/A
pGCT[RBM7-3F-mAID] HYG	This study	N/A
pGCT[RBM7-3F-mAID] NEO	This study	N/A
pGCT[ZFC3H 1-3F-mAID] HYG	This study	N/A
pGCT[ZFC3H 1-3F-mAID] NEO	This study	N/A
pGCT[MPP8-3F-mAID] HYG	This study	N/A
pGCT[MPP8-3F-mAID] NEO	This study	N/A
pGCT[TASOR-3F-mAID] HYG	This study	N/A
pGCT[TASOR-3F-mAID] NEO	This study	N/A
pGCT[MTR4-3F-mAID] HYG	This study	N/A
pGCT[MTR4-3F-mAID] NEO	This study	N/A
pGCT[MTR4-3F] HYG	This study	N/A
pGCT[MTR4-3F] NEO	This study	N/A
pGCT[ZCCHC8-3F] HYG	This study	N/A
pGCT[ZCCHC8-3F] NEO	This study	N/A
pGCT[RBM7-3F] HYG	This study	N/A
pGCT[RBM7-3F] NEO	This study	N/A
pGCT[TASOR-3F] HYG	This study	N/A
pGCT[TASOR-3F] NEO	This study	N/A
pBAC[MYC-ZCCHC8_1-709] BLAST	This study	N/A
pBAC[MYC-ZCCHC8_1-660] BLAST	This study	N/A
pBAC[MYC-ZCCHC8_1-337] BLAST	This study	N/A
pBAC[MYC-ZCCHC8_1-249] BLAST	This study	N/A
pBAC[MYC-ZCCHC8_1-153] BLAST	This study	N/A
pBAC[MYC-ZCCHC8_1-83] BLAST	This study	N/A
pBAC[MYC-ZCCHC8_214-709] BLAST	This study	N/A
pBAC[MYC-ZCCHC8_250-709] BLAST	This study	N/A
pBAC[MYC-ZCCHC8_288-709] BLAST	This study	N/A

Software and algorithms		

R	N/A	https://www.r-project.org/
Python	N/A	https://www.python.org/
RStudio	N/A	https://www.rstudio.com/
CHOPCHOP (v3)	Labun et al., 2019	https://chopchop.cbu.uib.no/
Trim Galore (v0.4.4)	N/A	https://www.bioinformatics.babraham.ac.uk/projects/trim_galore/
STAR (v2.7.3a)	Dobin et al., 2013	https://github.com/alexdobin/STAR/
TElocal (v0.1.0)	Jin et al., 2015	https://github.com/mhammell-laboratory/TElocal
edgeR	McCarthy et al. 2012	https://bioconductor.org/packages/release/bioc/html/edgeR.html
deepTools (v2.5.3)	Ramírez et al., 2014	https://deeptools.readthedocs.io/en/develop/
BBMap (v37.62)	N/A	https://sourceforge.net/projects/bbmap/
SAMtools (v1.9)	Li et al., 2009	https://github.com/samtools/
UMI-tools (v1.0.1)	Smith et al., 2017	https://github.com/CGATOxford/UMI-tools
BEDtools (v2.29.2)	Quinlan and Hall, 2010	https://bedtools.readthedocs.io/en/latest/
DEseq2 (v1.28.0)	Love et al., 2014	https://bioconductor.org/packages/release/bioc/html/DESeq2.html
bedGraphToBigWig	Kent et al., 2010	https://anaconda.org/bioconda/ucsc-bedgraphtobigwig
MACS2 (v2.1.1)	Zhang et al., 2008	https://github.com/macs3-project/MACS
SparK (v2.6.2)	Kurtenbach and William Harbour, 2019	https://github.com/harbourlab/SparK
ImageJ (v1.51)	Schneider et al., 2012	https://imagej.nih.gov/ij/
AriaMx (v1.71)	Agilent	https://www.agilent.com/
Graphpad Prism (9.0.0)	Graphpad	https://www.graphpad.com/scientific-software/prism/
Aminode	Chang et al., 2018	http://www.aminode.org/search

## Highlights

- The NEXT complex targets transposable element (TE) RNAs
- NEXT, via its ZCCHC8 subunit, physically interacts with the HUSH complex
- The HUSH factor MPP8 is required to recruit NEXT to chromatin at TE loci
- HUSH and NEXT function regulate pA^+^ and pA^−^ RNAs, respectively
