# Supplementary material for: Chromatin modifier HUSH co-operates with RNA decay factor NEXT to restrict transposable element expression
Source: Mol Cell. Author manuscript; Available in PMC 2022 Sep 1. (PMC9433625; doi:10.1016/j.molcel.2022.03.004)
Supplement: 1 [file NIHMS1829753-supplement-1.pdf]

Molecular Cell, Volume *82*

Supplemental information

Chromatin modifier HUSH co-operates with RNA decay factor NEXT to restrict transposable element expression

William Garland, Iris Müller, Mengjun Wu, Manfred Schmid, Katsutoshi Imamura, Leonor Rib, Albin Sandelin, Kristian Helin, and Torben Heick Jensen

Supplemental Figure 1. The NEXT complex impacts TE RNA levels, Related to Figure 1

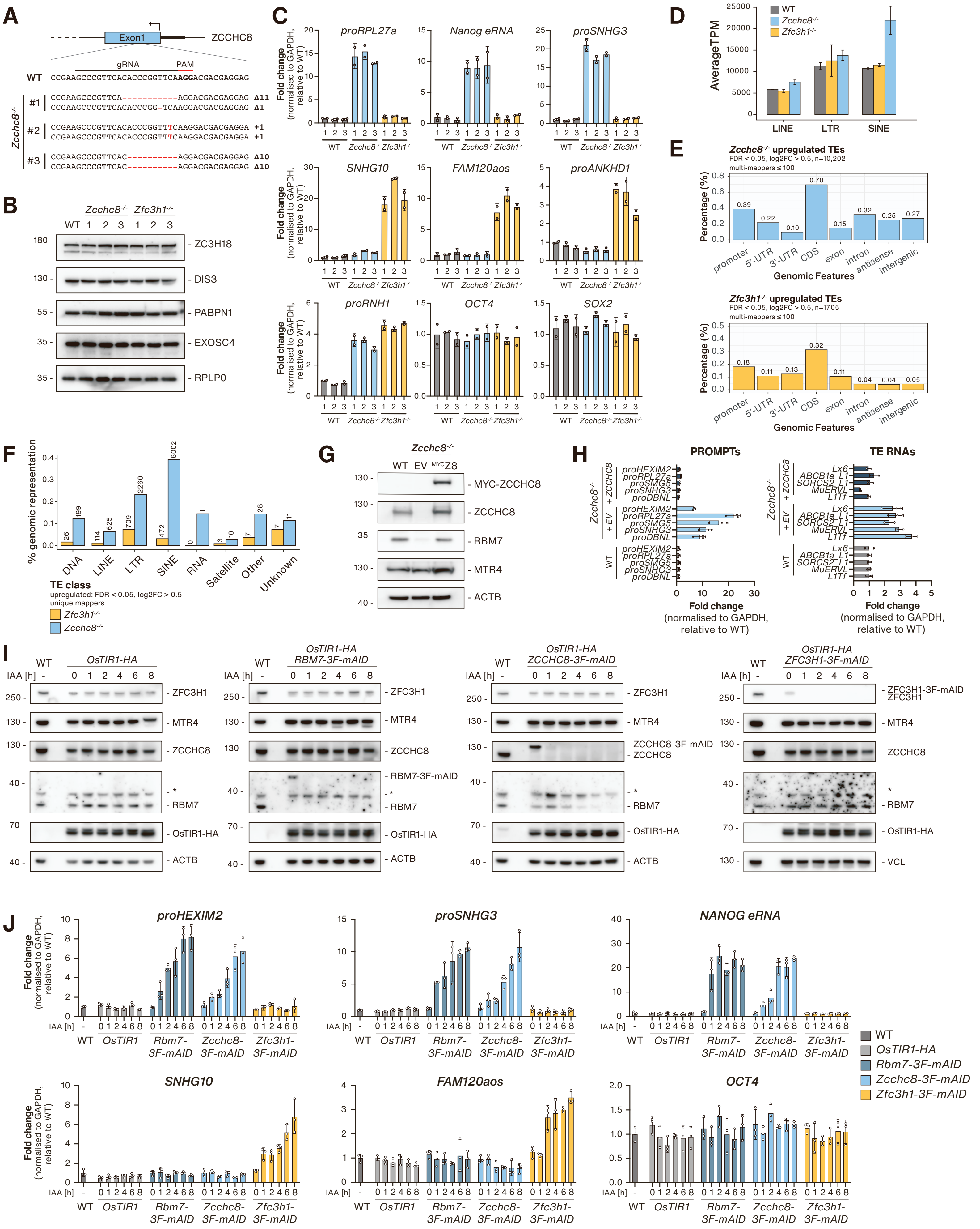

Supplemental Figure 1. The NEXT complex impacts TE RNA levels, Related to Figure 1.

(A) Genomic validation of CRISPR/Cas9-engineered *Zcchc8*$^{-/-}$ cell lines. PCR was carried out on genomic DNA isolated from three *Zcchc8*$^{-/-}$ cell lines derived from single cell clones. To distinguish individual alleles, amplicons were cloned into the pCR4 vector and sequenced. The gRNA sequence and PAM motif are highlighted on top with the WT sequence. Base pair deletions/insertions are indicated per allele (Δ/+n) **(B)** Western blotting analysis as in Figure 1B. Blots were probed with antibodies against the indicated exosome related proteins and RPLP0 as a loading control. **(C)** RT-qPCR analysis of total RNA isolated from WT, *Zcchc8*$^{-/-}$ and *Zfc3h1*$^{-/-}$ cell lines. Amplicons were designed against NEXT (*proRPL27a, Nanog eRNA, proSNHG3*), PAXT (*SNHG10, FAM120aos, proANKHD1*) or NEXT/PAXT (*proRNH1*) targets along with *OCT4* and *SOX2* mRNAs. WT samples were from 3 biological replicates whereas *Zcchc8*$^{-/-}$ and *Zfc3h1*$^{-/-}$ samples were from three independent clonal cell lines. Results shown were normalised to *GAPDH* mRNA relative to the average of WT samples and data was displayed as in Figure 1H. **(D)** Bar plots of average normalised read counts from WT, *Zcchc8*$^{-/-}$ and *Zfc3h1*$^{-/-}$ RNAseq samples mapping to retrotransposon classes (LINE, LTR and SINE). Bars represent the average transcripts per million (TPM) values from three replicates, with error bars denoting the standard deviation. **(E)** Genomic features of upregulated TE RNAs (log2FC > 0.5, FDR < 0.05) from *Zcchc8*$^{-/-}$ (n=10202) or *Zfc3h1*$^{-/-}$ (n=1705) samples. Bars depict the total percentage of upregulated TE RNAs relative to their genomic representation and stratified by their genomic location as based on Gencode annotations **(F)** As in Figure 1D but for uniquely mapped RNAseq data. **(G)** Western blotting analysis of WT control or *Zcchc8*$^{-/-}$ cells stably integrated with an either empty vector (EV) or a MYC-ZCCHC8 expressing construct. Membranes were probed with antibodies against MYC, ZCCHC8, RBM7, MTR4 and Actin (ACTB) as a loading control. **(H)** RT-qPCR analysis of indicated PROMPTs (left) or TE RNAs (right) from total RNA isolated from cells from (G). Data representation as in (C) **(I)** Western blotting analysis showing depletion kinetics of mAID-tagged proteins in OsTIR1-HA expressing cell lines.

Samples from control, *Rbm7-3F-mAID*, *Zcchc8-3F-mAID* and *Zfc3h1-3F-mAID* cell lines were taken at the indicated time points (hours) following exposure to IAA. WT samples were included on each blot for comparison. Membranes were probed with antibodies against ZFC3H1, MTR4, ZCCHC8, FLAG and HA as indicated. Actin (ACTB) or Vinculin (VCL) antibodies were used as loading controls. Non-specific bands are indicated with a star (*). **(J)** RT-qPCR analysis of NEXT and PAXT targets from total RNA isolated from cells from (I). *OCT4* mRNA was used as a control. Cell lines are represented as colours as indicated in the key. Values were normalised to *GAPDH* mRNA and the WT samples. Data representation as in Figure 1H.

Supplemental Figure 2. A physical and functional connection between the NEXT and HUSH complexes, Related to Figure 2

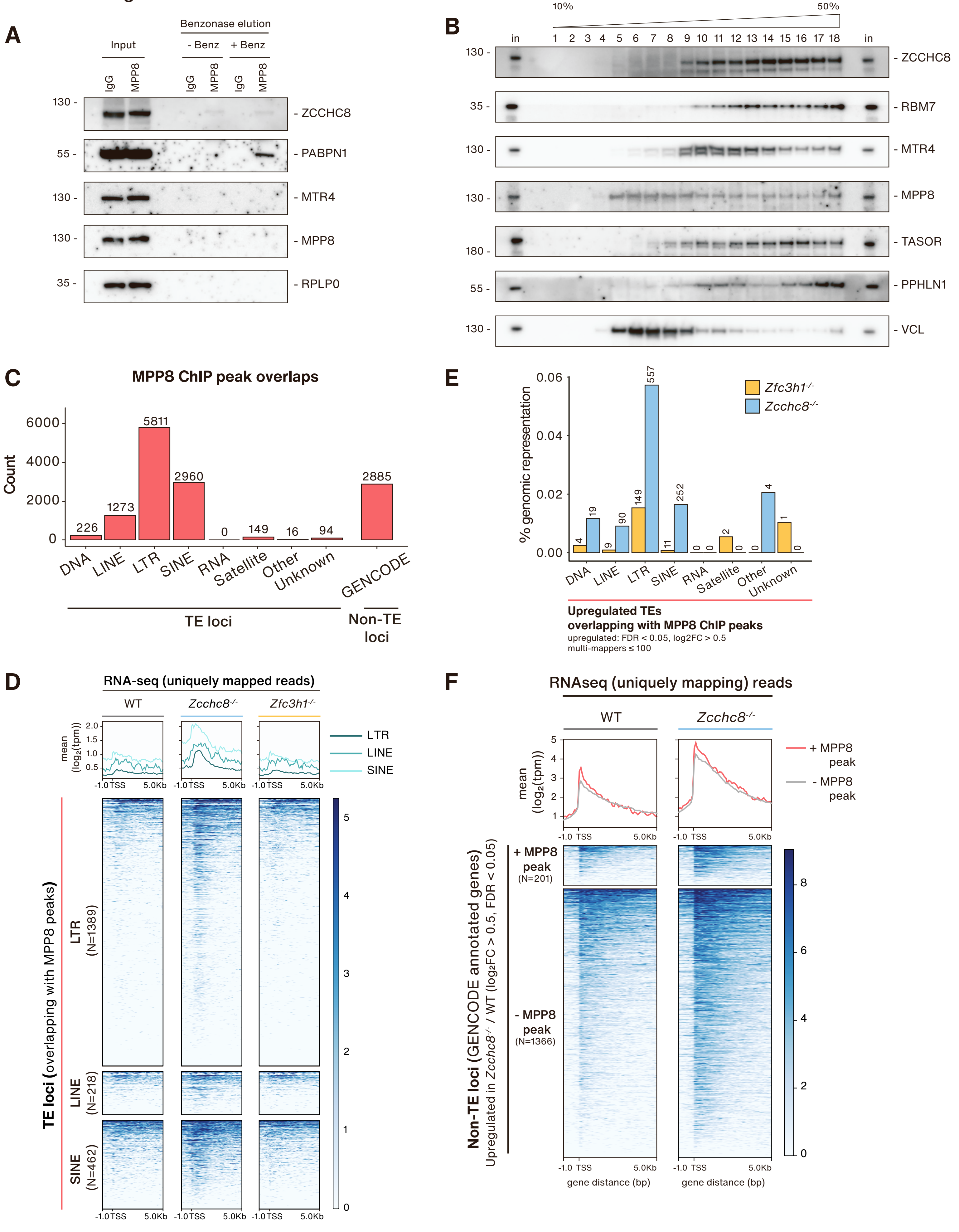

Supplemental Figure 2. A physical and functional connection between the NEXT and HUSH complexes, Related to Figure 2.

(A) Western blotting analysis of input and Benzonase elution samples from the experiment shown in Figure 2G. Following IgG or MPP8 IPs, bead-bound proteins were either mock- or Benzonase-treated to release interactions mediated by nucleic acids. Input and Benzonase elution samples were probed with antibodies against ZCCHC8, PABPN1, MTR4, MPP8 and RPLP0 as an input loading control. **(B)** Western blotting analysis of WT cell lysate following its sedimentation through 10-50% glycerol gradients and fractionation. Input (in) samples prior to sedimentation were loaded in the first and last lanes for comparison. Membranes were probed with antibodies against indicated NEXT and HUSH related proteins. Vinculin (VCL) was used as a marker for low molecular weight complexes. **(C)** Bar plots of MPP8 ChIP peaks overlapping with TE (stratified by TE class) or non-TE (GENCODE) -loci (x axis). Overlaps with GENCODE annotations do not exclude TE loci located within the genes. The y axis shows the count of loci with absolute numbers indicated above each bar. **(D)** Metagene (upper) and heatmap (lower) profiles of uniquely mapped RNA-seq reads from WT, *Zcchc8*$^{-/-}$ and *Zfc3h1*$^{-/-}$ samples at MPP8-peak regions overlapping with single TE loci. TEs, defined by Repeatmasker annotations were stratified into LTR, LINE and SINE classes with the number of elements indicated in parentheses. A region window of -1Kb to + 5Kb around the TE TSSs is displayed. **(E)** Bar plots of upregulated RNAs in *Zcchc8*$^{-/-}$ and *Zfc3h1*$^{-/-}$ samples at TE loci, that overlap with MPP8 ChIP peaks. Values show the percentage of genomic representation in each TE class, with the absolute numbers above each bar. **(F)** Metagene (upper) and heatmap (lower) profiles of RNA-seq reads from WT and *Zcchc8*$^{-/-}$ samples overlapping GENCODE annotated genes upregulated ($log_2FC > 0.5$, $FDR < 0.05$) in *Zcchc8*$^{-/-}$ vs. WT samples. Panels were stratified into genes that are overlapping (+) or non-overlapping (-) with MPP8 ChIP peaks.

Supplemental Figure 3. ZCCHC8 bridges the interaction between NEXT and HUSH, Related to Figure 3

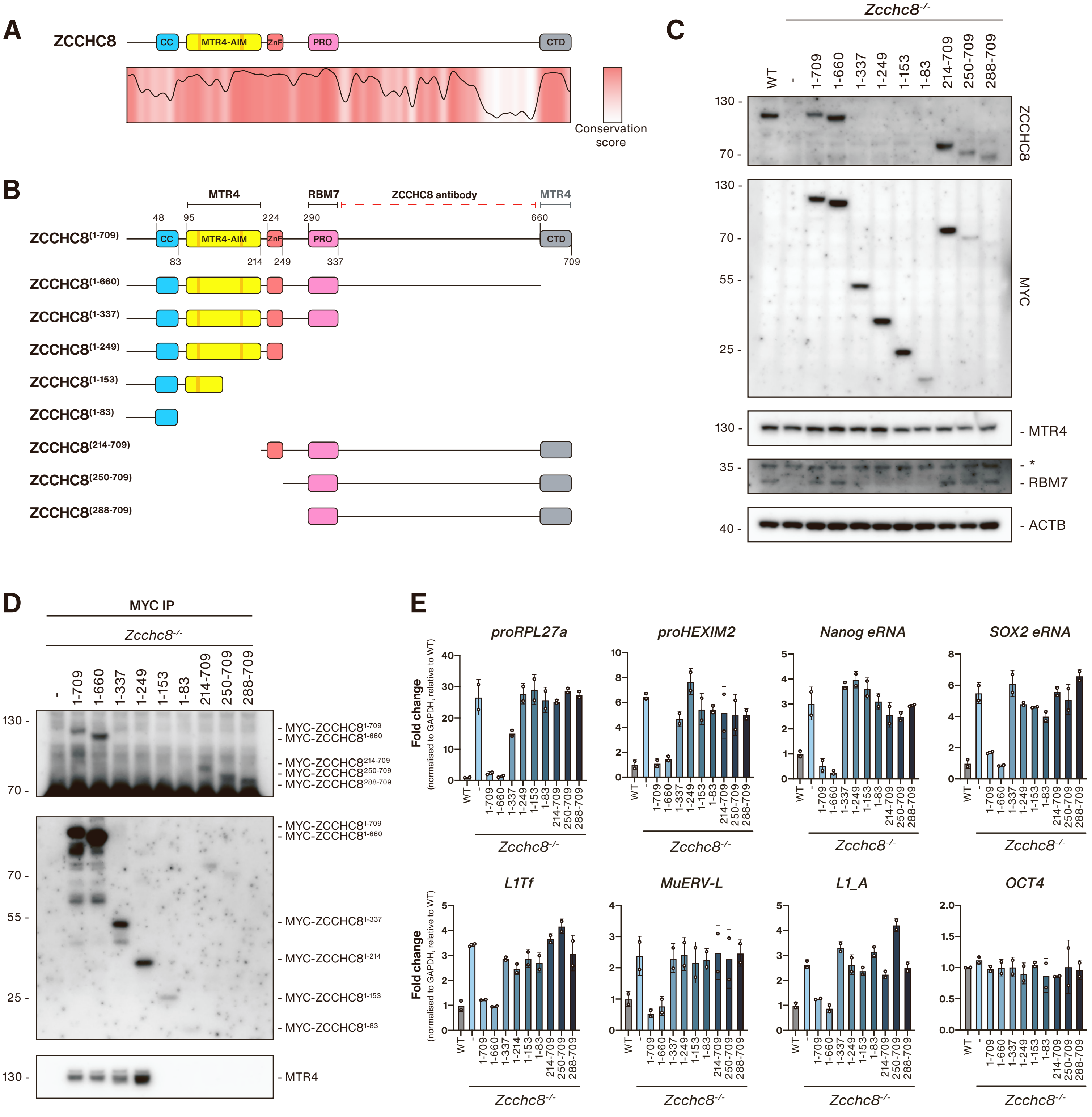

Supplemental Figure 3. ZCCHC8 bridges the interaction between NEXT and HUSH, Related to Figure 3.

(A) Schematic representation of the ZCCHC8 domain organisation with a heatmap depicting regions of conservation. Domain boundaries are adapted from studies of the human ZCCHC8 protein (Falk et al., 2016; Lingaraju et al., 2019; Puno and Lima, 2018). Heatmap data was generated using evolutionary constrained regions (ECR) data from the Aminode webtool (Chang et al., 2018). A trace line is used to indicate the heatmap intensity. **(B)** Schematic representation of the ZCCHC8 fragments generated in this study. Domain boundary amino acid residues are depicted on the full length (1-709) representation. Known binding domains for MTR4 and RBM7 are indicated. The location of two MTR4 arch interacting motifs (AIMs) are indicated as orange lines within the MTR4-AIM domain (Lingaraju et al., 2019). The recognised epitope of the ZCCHC8 antibody used this in study is depicted as a red-dashed line. **(C)** Western blotting analysis of lysates from WT or *Zcchc8*$^{-/-}$ cells stably expressing MYC-tagged ZCCHC8 fragments from (B). Samples were probed with antibodies against ZCCHC8, MYC, MTR4 and RBM7. Non-specific bands are shown as an asterisk (*). Actin (ACTB) was used as a loading control. **(D)** Western blotting analysis of MYC IP samples from *Zcchc8*$^{-/-}$ cells stably expressing MYC-tagged ZCCHC8 fragments as shown in (B) and (C). IP samples were probed with antibodies against ZCCHC8, MYC and MTR4. **(E)** RT-qPCR analysis of known NEXT targets (*proRPL27a*, *proHEXIM2*, *Nanog eRNA*, *Sox2 eRNA*) and TE RNAs (*L1Tf*, *MuERV-L*, *L1_A*) from total RNA isolated from equivalent samples described in (C). *OCT4 mRNA* was used as a control. Data representation as described in Figure 1H.

Supplemental Figure 4. NEXT recruitment to chromatin at HUSH-bound loci depends on MPP8, Related to Figure 4

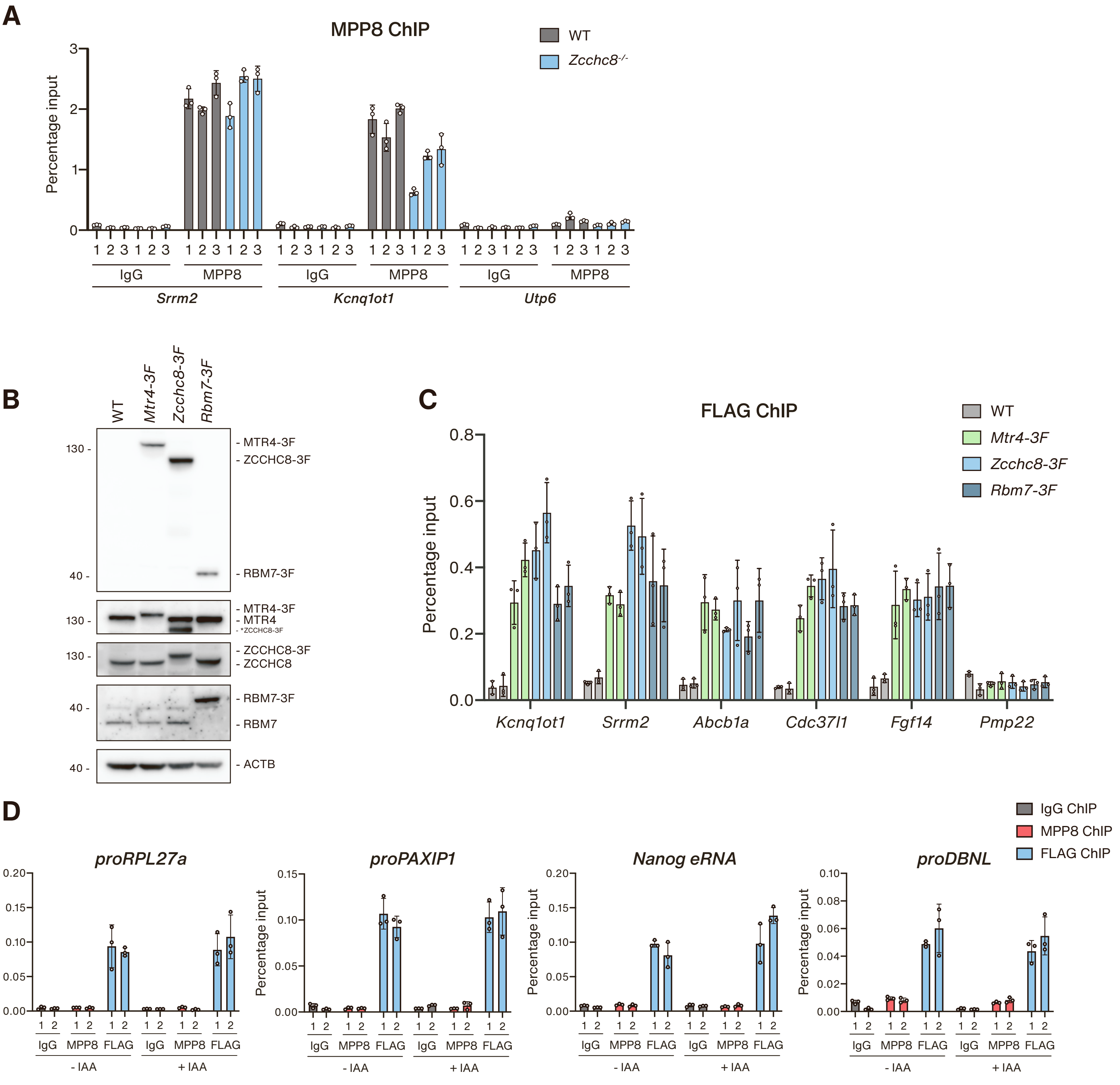

Supplemental Figure 4. NEXT recruitment to chromatin at HUSH-bound loci depends on MPP8, Related to Figure 4.

(A) qPCR analysis of IgG and MPP8 ChIPs at MPP8 bound loci (*Srrm2, Kcnq1ot1*) or control regions not bound by MPP8 (*Utp6*) from three biologically independent WT or *Zcchc8*$^{-/-}$ replicate samples. WT and *Zcchc8*$^{-/-}$ samples were from 3 biological replicates and 3 clonal cell lines, respectively. Data representation as in Figure 4B. **(B)** Western blotting analysis of lysates from WT, *Mtr4-3F*, *Zcchc8-3F* and *Rbm7-3F* cells. Membranes were probed with antibodies against FLAG, MTR4, ZCCHC8, RBM7 and Actin (ACTB) as a loading control. **(C)** qPCR analysis of FLAG ChIPs at MPP8 bound- (*Kcnq1ot1*, *Srrm2*, *Abcb1a*, *Cdc37l1*, *Fgf14*) and unbound control (*Pmp22*)-loci from two biological replicates of WT, *Mtr4-3F*, *Zcchc8-3F* and *Rbm7-3F* cells. Data shown represent percentage input values with error bars denoting the SD and individual data values marked as points. **(D)** qPCR analysis of IgG, MPP8 and FLAG ChIPs at NEXT-sensitive loci (*proRPL27a*, *proPAXIP1*, *Nanog eRNA, proDBNL*) from *Mpp8-mAID Zcchc8-3F OsTIR1-HA* samples as described in Figures 4F and 4G.

Supplemental Figure 5. NEXT and HUSH suppress non-polyadenylated and polyadenylated TE RNAs, respectively, Related to Figure 5

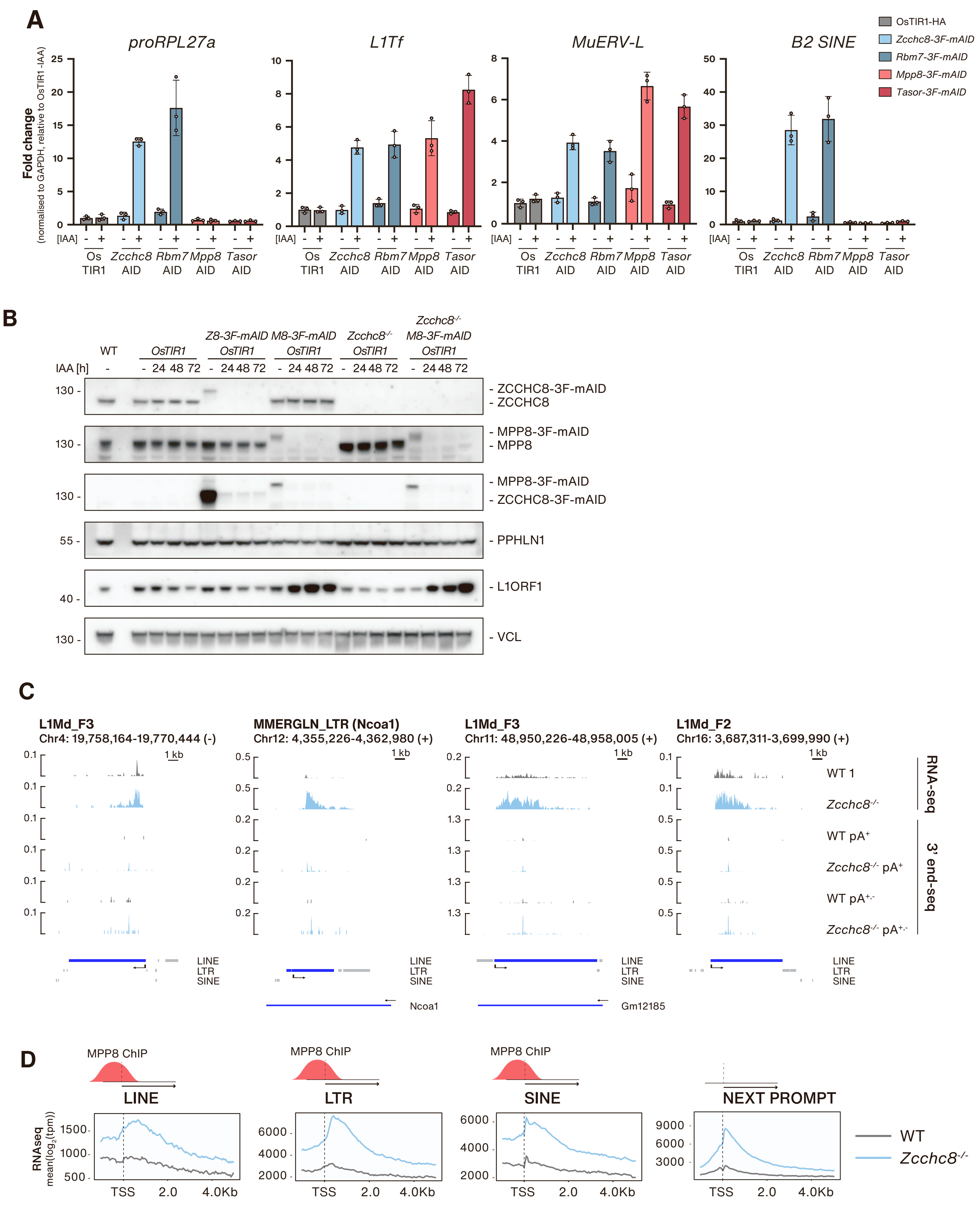

Supplemental Figure 5. NEXT and HUSH suppress non-polyadenylated and polyadenylated TE RNAs, respectively, Related to Figure 5

(A) RT-qPCR analysis of a NEXT target (*proRPL27a*) or TE transcripts (*L1Tf, MuERV-L*, *B2 SINE*) from total RNA isolated from control (*OsTIR1*), *Zcchc8-3F-mAID*, *Rbm7-3F-mAID, Mpp8-3F-mAID* or *Tasor-3F-mAID* cells either mock- or IAA-treated (72h). Data were normalised to GAPDH mRNA and plotted relative to the control – IAA sample. The average of 3 biological replicates with error bars denoting the SD and individual values plotted as points is shown. **(B)** Western blotting analysis of lysates from WT, *OsTIR1*, *Zcchc8-3F-mAID*, *Mpp8-3F-mAID, Zcchc8$^{-/-}$* or *Zcchc8$^{-/-}$ Mpp8-3F-mAID* samples. Control (*OsTIR1*) and -mAID expressing cell lines were either mock treated or grown in the presence of IAA for the indicated times (24, 48, 72 hours). Membranes were probed with antibodies against ZCCHC8, MPP8, FLAG, PPHLN1, L1ORF1 and Vinculin (VCL) as a loading control. **(C)** Genome browser tracks of upregulated TE transcripts from RNA-seq and 3'end-seq data generated from WT and *Zcchc8$^{-/-}$* cell lines. RNA-seq tracks show one replicate of each sample and display reads covering the entire expressed unit. 3'end-seq tracks show the average of three biological replicate samples, including either pA^{+} or pA$^{+,-}$ libraries and display reads from RNA 3' ends only. TE annotations and gene models are displayed as in Figure 1F. Genome co-ordinates include strand information for forward (+) and reverse (-) strands as indicated. **(D)** Metagene analysis of uniquely mapped RNA-seq reads from WT and *Zcchc8$^{-/-}$* datasets at TE loci overlapping with MPP8 ChIP peaks from Fig S2E or NEXT-sensitive PROMPT loci. TE loci were stratified into LINE, LTR and SINE classes with a region window of -1Kb to + 5Kb around the TE TSSs displayed.

Supplemental tables

Table S1: sgRNA oligonucleotides, Related to STAR methods

NAME	SEQUENCE
Zcchc8_CRISPR_KO_2_F	CACCGGCCCGTTCACACCCGGTTCA
Zcchc8_CRISPR_KO_2_R	AAACTGAACCGGGTGTGAACGGGCC
Zcchc8_CRISPR_KI_F	CACCGAAAGACTTGTGAGTGAGCCC
Zcchc8_CRISPR_KI_R	AAACGGGCTCACTCACAAGTCTTTCC
Mpp8_CRISPR_KI_F	CACCGAGAACCGGCCTCTTTTCAGC
Mpp8_CRISPR_KI_R	AAACGCTGAAAAGAGGCCGGTTCTCC
Tasor_CRISPR_KI_F	CACCGCTTTTAGCATCGGATTATC
Tasor_CRISPR_KI_R	AAACGATAATCCGATGCTAAAAGCC
Zfc3h1_CRISPR_KI_F	CACCGATTAGGCTGATCCCTGGCTA
Zfc3h1_CRISPR_KI_R	AAACTAGCCAGGGATCAGCCTAATCC
Rbm7_CRISPR_KI_F	CACCGTTCCAAGGACACTGTTTACA
Rbm7_CRISPR_KI_R	AAACTGTAAACAGTGTCCTTGGAACC
Mtr4_CRISPR_KI_F	CACCGTCAGTTGCAATCAGACAGG
Mtr4_CRISPR_KI_R	AAACCCTGTCTGATTGCAACTGACC

Table S2: RTqPCR primers, Related to STAR methods

NAME	FORWARD	REVERSE
Abcb1a_L1	GACCAGGCGGAAGCACAGAG	CGGGTCTCGGACCAAGATGG
B2 SINE	CCAGAGTTCAAATCCCAGCAAC	ACTACCCCAACTGTGGATGAG
Fam120aos	CAGGAGTGACCCATCCATCG	CCATCAAGGCCCTAAGCGC
Gapdh	TTGATGGCAACAATCTCCAC	CGTCCCGTAGACAAAATGGT
L1_A	GGATTCCACACGTGATCCTAA	TCCTCTATGAGCAGACCTGGA

L1_Md_F2_2	CACCAGCCACGCGATCTTAAG	CGGGAAAATGGTCACCTGCC
L1_Md_F2_4	CCCAAAGCGAGGCAACACTTG	GTTGTGCCGGTGTTCTCTATGG
L1_Md_F2_6	GAACCTCACGGGACCTTCTCC	CCACGTGAATGTTGGCTTTCC
L1_Md_F2_8	CTCAGAAGATGGAAAGACCTCCC	GGAATTTTGATGGGGATAGCATTG
L1_Md_F2_9	GGTGACAGCAGATGCTTGCG	TTTCTGAGGAACCGCCAGAC
L1_Md_T_1	GCAGTGGTCGCCATCTTGG	CTGTCCTCCGGTCCGGAAG
L1_Md_T_4	CCAGGACCAGACGGGTTTAG	GTGTTCTTGGATTCGGTTAGCGAG
L1_Md_T_5	CGCCTCACACAATAATAGTGGGAG	CTTCTGTTAGTTTCAGTGTGTCCC
L1_Md_T_6	CCCAGAAATGAACCCACACAC	GCTTTTCCCCACTTTCTCCTC
L1_Md_T_7	CCACCTCACACCAGTGAGAATG	CCGCCAGACTGATTTCCAGA
L1Tf	GCCTAAGCCACAGCAGCA	GCTGTCAGGTTCTCTGGCG
Lx6	GGGGCACCATTTAGGGGTTG	GGTTTTACCCGAGGTCTCTGGG
MuERV-L	GGCTGCTCTACCACTTGGAC	TCAGCCACAGACACCTCAAG
Nanog eRNA	GTCTGCCAGCGATTCTTTTC	GTCTGAAGCCGGTTCACTTC
Oct4	CAGCAGATCACTCACATCGCCA	GCCTCATACTCTTCTCGTTGGG
proAnkhd1	CTTGGTCCTGAGAACTGTCCG	CGCTCCGAGGTCACTCAACA
proDbnl	GGCGCCTCGGAAATTTCATC	CAGGGCTTCTCTGCCTAGCC
proHexim2	GATCCTGAGGCCGAGGTCTG	GCGAGCCAGGGTCTCCAAC
proRnh1	GGTGCTCTTAGGGGTGCTTG	GAGGCCTTGGCTCCGAAAC
proRpl27a	CGTCGGAGTGCACTGTTCTT	GAAGTCTTGCCGATGCTCTG
proSmg5	CTGAGCGTTCTAGCGCAGG	TTAGCCGCTGAGTCATCTCG
proSnhg3	AGGTGTTGGCGGTCTTAGCG	CTAAGCCACCTCTCAGGCCC
Snhg10	GCCTTCCATGCCTCACCG	GAATCAGAGGATCCTGCAAG
Sorcs2_L1	CGCCAGAGAACCTGACAGCT	TCTGTGCTTCCGCCTGTTCC
Sox2	AACGGCAGCTACAGCATGATGC	CGAGCTGGTCATGGAGTTGTAC
Sox2 eRNA	CACCTCCGAACCCAGGTTTT	GCTCAGAGTCTCCGCTTCTC

Table S3: ChIP qPCR primers, Related to STAR methods

NAME	FORWARD	REVERSE
Kcnq1ot1	GGACTAGCTGGCAAACCTTCA	CCAGGCCAATCCAAAGCATTC
Srrm2	GCTCGAAGAGGTTCCAGGTC	CTGGAGCGGATTTCTGGTGA
Pmp22	AGCCACCATGCTCCTACTCT	GAAGAGCAACACTAGCACCG
Utp6	TCTATGGCCTTACCCACTGC	TGACACGTTTCTGCTTCCAG
Cdc37l1	ATTTCCTCCAACAAAGCCACATC	TGTGGTGATTTGAATAGGTATGGCC
Ncoa1	GAATAGCTCAGGGCTGGTCTG	CATGGCTAACCCACCTATTCAC
Fgf14	GAGACTGAGCTTGTCCTTG	TCTTTTGGGGAAGTCTAGGTAC
Abcb1a	GACCAGGCGGAAGCACAGAG	CGGGTCTCGGACCAAGATGG
proRPL27a_ChIP	CTCAGAGCATCGGCAAGACTT	GCATACCAGCCAGAGTCTGG
proPAXIP1_ChIP	GGCAGACCCTGGCTAATGG	GATGTCTGCCTGCTGAGCTATG
Nanog eRNA_ChIP	CACAATGGGCTGGACCCTTC	GTGTGTTCGAGGCTGGCAG
proDBNL_ChIP	CGGACATGATGGCTACGCC	CCTCTACGCCTAACACAGCAG
